# Durability analysis of the highly effective mRNA-1273 vaccine against COVID-19

**DOI:** 10.1093/pnasnexus/pgac058

**Published:** 2022-05-20

**Authors:** Arjun Puranik, Patrick J Lenehan, John C O'Horo, Colin Pawlowski, Abinash Virk, Melanie D Swift, Walter Kremers, A J Venkatakrishnan, Doug W Challener, Laura Breeher, Joel E Gordon, Holly L Geyer, Leigh Lewis Speicher, Venky Soundararajan, Andrew D Badley

**Affiliations:** nference, Cambridge, MA 02139, USA; nference, Cambridge, MA 02139, USA; Division of Infectious Diseases, Mayo Clinic, Rochester, MN 55902, USA; Division of Pulmonary and Critical Care Medicine, Mayo Clinic, Rochester, MN 55902, USA; nference, Cambridge, MA 02139, USA; Division of Infectious Diseases, Mayo Clinic, Rochester, MN 55902, USA; Division of Preventive, Occupational, and Aerospace Medicine, Mayo Clinic, Rochester, MN 55902, USA; Division of Biomedical Statistics, Mayo Clinic, Rochester, MN 55902, USA; nference, Cambridge, MA 02139, USA; Division of Infectious Diseases, Mayo Clinic, Rochester, MN 55902, USA; Division of Preventive, Occupational, and Aerospace Medicine, Mayo Clinic, Rochester, MN 55902, USA; Department of Family Medicine, Mayo Clinic Health System, Mankato, MN 56001, USA; Division of Hospital Internal Medicine, Mayo Clinic, Scottsdale, AZ 85259, USA; Division of General Internal Medicine, Mayo Clinic, Jacksonville, FL 32224, USA; nference, Cambridge, MA 02139, USA; nference Labs, Bengaluru, Karnataka 560017, India; Division of Infectious Diseases, Mayo Clinic, Rochester, MN 55902, USA; Department of Molecular Medicine, Mayo Clinic, Rochester, MN 55902, USA

**Keywords:** COVID-19, SARS-CoV-2, mRNA-1273, vaccine effectiveness, vaccine durability

## Abstract

COVID-19 vaccines are effective, but breakthrough infections have been increasingly reported. We conducted a test-negative case-control study to assess the durability of protection against symptomatic infection after vaccination with mRNA-1273. We fit conditional logistic regression (CLR) models stratified on residential county and calendar date of SARS-CoV-2 PCR testing to assess the association between the time elapsed since vaccination and the odds of symptomatic infection, adjusted for several covariates. There were 2,364 symptomatic individuals who had a positive SARS-CoV-2 PCR test after full vaccination with mRNA-1273 (“cases”) and 12,949 symptomatic individuals who contributed 15,087 negative tests after full vaccination (“controls”). The odds of symptomatic infection were significantly higher 250 days after full vaccination compared to the date of full vaccination (Odds Ratio [OR]: 2.47, 95% confidence interval [CI]: 1.19–5.13). The odds of non-COVID-19 associated hospitalization and non-COVID-19 pneumonia (negative control outcomes) remained relatively stable over the same time interval (Day 250 OR_Non-COVID Hospitalization_: 0.68, 95% CI: 0.47–1.0; Day 250 OR_Non-COVID Pneumonia_: 1.11, 95% CI: 0.24–5.2). The odds of symptomatic infection remained significantly lower almost 300 days after the first mRNA-1273 dose as compared to 4 days after the first dose, when immune protection approximates the unvaccinated state (OR: 0.26, 95% CI: 0.17–0.39). Low rates of COVID-19 associated hospitalization or death in this cohort precluded analyses of these severe outcomes. In summary, mRNA-1273 robustly protected against symptomatic SARS-CoV-2 infection at least 8 months after full vaccination, but the degree of protection waned over this time period.

Significance Statement COVID-19 vaccines are effective, but breakthrough infections do occur. It is important to evaluate how vaccine effectiveness changes over time in the context of newly evolved and highly distinctive SARS-CoV-2 lineages such as the Omicron variant. Here, we assess the durability of protection conferred by mRNA-1273 against symptomatic COVID-19 from January 2021 through January 2022. mRNA-1273 provided strong protection for at least 8 months after full vaccination, but its effectiveness did significantly wane during this period. This study highlights the importance of continuing to monitor the effectiveness and durability of both primary series and booster doses of COVID-19 vaccines.

## Introduction

The coronavirus disease-2019 (COVID-19) pandemic stimulated the most rapid cycle of vaccine development, testing, and deployment in history. The two mRNA vaccines authorized by the United States Food and Drug Administration (FDA), mRNA-1273 (Moderna) and BNT162b2 (Pfizer-BioNTech), both encode a prefusion stabilized form of the Spike glycoprotein of severe acute respiratory syndrome coronavirus 2 (SARS-CoV-2) (1,2). These vaccines showed over 90% efficacy in the prevention of symptomatic COVID-19 during large randomized trials (3, 4), and their effectiveness was corroborated by a multitude of studies in large health systems around the world (5–14). Recently, several real-world analyses have shown that mRNA-1273 conferred stronger protection against symptomatic disease than BNT162b2, which may be related to differences in the dosing or formulation of these vaccines (15–18).

Increasing reports of breakthrough infections (i.e. infections in fully vaccinated individuals) amidst the surge of the Delta variant starting in July 2021 raised questions about the durability of these vaccines, and these questions have only been augmented with the most recent global surge of the Omicron variant. After several studies demonstrated reductions in neutralizing antibody titers and protection against symptomatic SARS-CoV-2 infection several months after vaccination, the Centers for Disease Control and Prevention (CDC) recommended that individuals at high risk for infection or severe disease should receive an additional (booster) vaccine dose (19–29). However, relatively few studies have specifically analyzed the durability of mRNA-1273 (30) or even included individuals receiving this vaccine (20–22), while more studies have analyzed the durability of BNT162b2, perhaps due its wider use both in the United States and worldwide (31,32). It is thus critical to gather additional data to better understand the durability of protection against infection conferred by vaccination with mRNA-1273.

## Results

**Figure 1.**
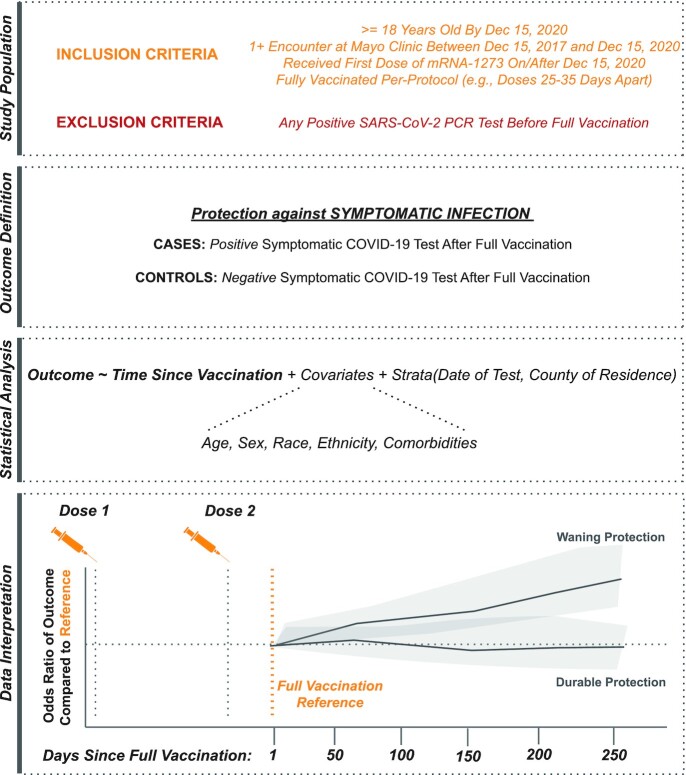
Schematic representation of study design. From top to bottom, (i) inclusion and exclusion criteria to define the population eligible for this test-negative analysis, (ii) definition of the clinical outcomes of interest, (iii) framework for statistical analysis, and (iv) schematic representation of data interpretation. (i) Individuals were included if they were at least 18 years old, had a record of at least one encounter at the Mayo Clinic in the 3 years prior to the study start date, and were fully vaccinated per-protocol with mRNA-1273 (with the first dose administered on or after 2020 December 15), and underwent at least one symptomatic SARS-CoV-2 PCR test after the date of full vaccination. Individuals were excluded if they received a positive PCR test prior to their date of full vaccination. (ii) The outcome was defined as symptomatic SARS-CoV-2 infection, and cases and controls were defined accordingly. (iii) Conditional logistic regression was used to assess the potential relationship between the odds of experiencing symptomatic infection and time since vaccination, while accounting for other clinical and demographic covariates. (iv) The odds of symptomatic infection were assessed over time after full vaccination (modeled as a linear spline) relative to the odds at the date of full vaccination, which is expected to correspond to maximal vaccine-mediated protection. An increase in the odds ratio with time since vaccination would be interpreted as evidence for waning protection, while a consistent odds ratio over (relative) time would be interpreted as durable protection.

Of 122,710 individuals who received two doses of mRNA-1273, 25 to 35 days apart, with no evidence of SARS-CoV-2 infection before reaching their date of full vaccination (14 days after the second dose), 20,165 subsequently underwent symptomatic testing and were eligible for inclusion in the test-negative case-control analysis (Figure 1; Figure S1 and Table S1, Supplementary Material).

In the primary analysis, the conditional logistic regression (CLR) model was stratified on residential county and the calendar date of testing in order to control for geographic and temporal differences in community exposure levels, SARS-CoV-2 variant prevalence, and implementation of nonpharmaceutical interventions (NPIs) such as masking and social distancing. There were 2,364 cases and 15,087 controls that contributed to analyzable strata for this analysis (i.e. strata with at least one case and at least one control; Table S1, Supplementary Material). Demographic and clinical characteristics of cases and controls were generally similar to each other and to the underlying population of fully vaccinated individuals (Table S1, Supplementary Material).

**Figure 2.**
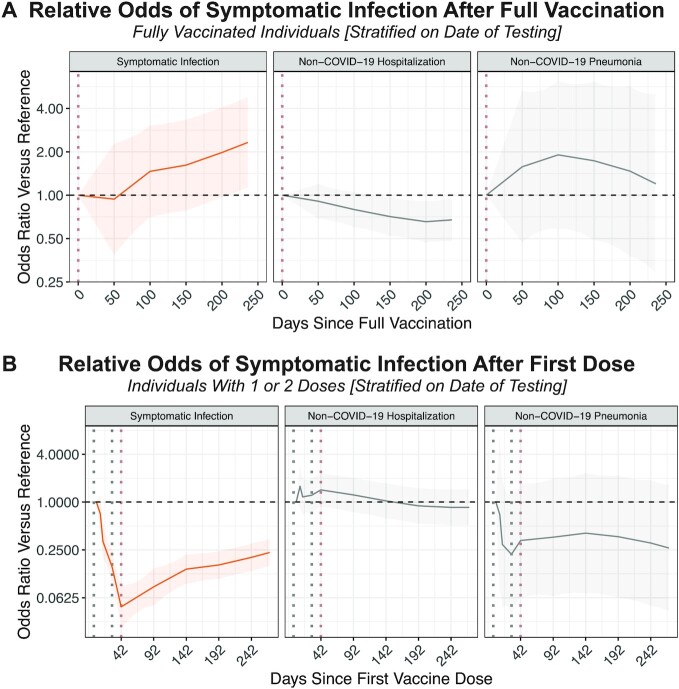
Relationship between time since vaccination and the adjusted odds of experiencing each outcome of interest. (A) Adjusted odds ratios of outcomes over time relative to the date of full vaccination, defined as 14 days after the second dose of mRNA-1273. Odds ratios correspond to the odds of experiencing the outcome at a given time *t* divided by the odds of experiencing that outcome at the reference date (*t* *=* 0 days since full vaccination), adjusted for several demographic and clinical covariates via CLR. (B) Adjusted odds ratios of outcomes over time relative to 4 days after the first mRNA-1273 dose, which is intended to approximate the unvaccinated status. Odds ratios correspond to the odds of experiencing the outcome at a given time *t* divided by the odds of experiencing that outcome at the reference date (*t* *=* 4 days since first vaccine dose), again adjusted for demographic and clinical covariates via CLR. The outcomes are symptomatic SARS-CoV-2 infection (shown in orange), non-COVID-19 associated hospitalization, and non-COVID-19 pneumonia (negative control outcomes shown in gray). For both panels, the CLR was stratified on residential county and the date of SARS-CoV-2 PCR testing.

Adjusted for age, sex, race, ethnicity, and comorbidities, the odds of symptomatic infection remained stable for several months but increased by 250 days after full vaccination (Odds Ratio (OR)_50 Days_: 0.94, 95% CI: 0.39–2.28; OR_100 Days_: 1.46, 95% CI: 0.70–3.04; OR_150 Days_: 1.62, 95% CI: 0.78–3.35; OR_200 Days_: 1.98, 95% CI: 0.96–4.07; OR_250 Days_: 2.47, 95% CI: 1.19–5.13; Figure 2A and Table 1). Among the other covariates, Asian race and the prior diagnosis of either cardiovascular disease or chronic pulmonary disease were associated with slightly lower odds of symptomatic infection after full vaccination (Table S2, Supplementary Material). The odds of experiencing non-COVID-19 hospitalization or pneumonia (negative controls) remained relatively stable over time (Figure 2A and Table 1).

**Table 1.** Adjusted odds of experiencing each outcome of interest at defined dates after full vaccination or the first vaccine dose. The outcomes of interest are symptomatic SARS-CoV-2 infection, non-COVID-19 associated hospitalization (negative control), and non-COVID-19 pneumonia (negative control). The top panel shows adjusted odds of experiencing each outcome in 50-day intervals after the date of full vaccination (i.e. 14 days after the second dose). The bottom panel shows adjusted odds of experiencing each outcome at relevant time points after the first vaccine dose; the expected date of second dose administration is 28 days after the first dose, and the expected date of full vaccination is 42 days after the first dose. On the left side, results are shown for the CLR models stratified on residential county and the calendar date of SARS-CoV-2 testing. On the right side, results are shown for the CLR models stratified on residential county, the date of full vaccination, and the 7-day trailing county-level COVID-19 incidence on the date of the SARS-CoV-2 PCR test.

	Stratified on date of testing			Stratified on date of vaccination		
	Symptomatic infection	Non-COVID-19 hospitalization	Non-COVID-19 pneumonia	Symptomatic infection	Non-COVID-19 hospitalization	Non-COVID-19 pneumonia
**Days since full vaccination**	*N* = 2,364	*N* = 3,385	*N* = 273	*N* = 2,388	*N* = 3,597	*N* = 288
0	1 (Reference)	1 (Reference)	1 (Reference)	1 (Reference)	1 (Reference)	1 (Reference)
50	0.94 (0.39–2.28)	0.91 (0.69–1.2)	1.57 (0.47–5.27)	1.77 (0.67–4.65)	1.05 (0.83–1.32)	1.96 (0.72–5.34)
100	1.46 (0.7–3.04)	0.8 (0.6–1.06)	1.91 (0.58–6.22)	6.92 (2.85–16.83)	0.88 (0.71–1.1)	1.55 (0.63–3.8)
150	1.62 (0.78–3.35)	0.71 (0.52–0.97)	1.73 (0.49–6.16)	8.78 (3.54–21.73)	0.7 (0.54–0.91)	1.16 (0.41–3.3)
200	1.98 (0.96–4.07)	0.65 (0.47–0.91)	1.47 (0.38–5.73)	13.16 (5.3–32.68)	0.68 (0.51–0.92)	1.63 (0.51–5.22)
250	2.47 (1.19–5.13)	0.68 (0.47–1)	1.11 (0.24–5.2)	23.01 (9.11–58.12)	0.78 (0.56–1.09)	1.12 (0.3–4.16)
**Days since first dose**	*N* = 2,684	*N* = 4,025	*N* = 310	*N* = 2,774	*N* = 4,286	*N* = 329
4	1 (Reference)	1 (Reference)	1 (Reference)	1 (Reference)	1 (Reference)	1 (Reference)
10	0.71 (0.42–1.21)	1.58 (0.84–3)	0.7 (0.07–6.87)	0.6 (0.36–0.98)	1.51 (0.88–2.59)	1.08 (0.17–6.84)
14	0.32 (0.2–0.51)	1.16 (0.69–1.95)	0.29 (0.05–1.89)	0.28 (0.17–0.44)	1.09 (0.71–1.69)	0.42 (0.09–1.99)
28	0.15 (0.09–0.25)	1.22 (0.74–2.02)	0.22 (0.04–1.31)	0.1 (0.06–0.17)	1.3 (0.85–2)	0.62 (0.13–2.91)
42	0.05 (0.03–0.09)	1.43 (0.87–2.32)	0.33 (0.06–1.74)	0.05 (0.03–0.09)	1.52 (1.01–2.28)	0.64 (0.15–2.62)
92	0.09 (0.05–0.15)	1.23 (0.75–2.03)	0.36 (0.07–1.99)	0.08 (0.04–0.14)	1.66 (1.1–2.49)	1.13 (0.28–4.51)
142	0.14 (0.09–0.22)	1.04 (0.62–1.72)	0.41 (0.07–2.32)	0.28 (0.18–0.44)	1.44 (0.95–2.18)	1 (0.25–3.94)
192	0.16 (0.11–0.24)	0.9 (0.54–1.5)	0.37 (0.06–2.15)	0.39 (0.25–0.6)	1.23 (0.8–1.88)	0.92 (0.23–3.74)
242	0.2 (0.14–0.3)	0.86 (0.51–1.45)	0.31 (0.05–1.89)	0.58 (0.38–0.9)	1.23 (0.79–1.9)	1.07 (0.25–4.63)
292	0.26 (0.17–0.39)	0.86 (0.5–1.49)	0.24 (0.03–1.65)	0.99 (0.63–1.55)	1.43 (0.9–2.28)	0.81 (0.17–3.86)

By stratifying on the calendar time of testing, individuals with small or large gaps between vaccination and testing within any given stratum were inherently vaccinated during different phases of the rollout. This would result in the comparison of individuals who likely differed in comorbidities, occupational risks of exposure to SARS-CoV-2, willingness to be vaccinated, and/or likelihood of engaging with the healthcare system. To address this, we modified the CLR to instead stratify on the date of vaccination and the county-level COVID-19 incidence at the time of PCR testing (see Methods). This yielded 2,388 positive symptomatic tests (cases) and 20,978 negative symptomatic tests (controls) contributing to analyzable strata (Table S1, Supplementary Material). With this modified approach, the adjusted odds of symptomatic infection at 150, 200, and 250 days after full vaccination were 8.78 (95% CI: 3.54–21.73), 13.2 (95% CI: 5.30–32.7), and 23.0 (95% CI: 9.11–58.1) times higher than at the date of full vaccination, respectively (Figure S2A, Supplementary Material; Table 1). Asian race and the prior diagnosis of cardiovascular disease were associated with modestly lower odds of symptomatic infection, while male sex was associated with slightly higher odds (Table S3, Supplementary Material).

To contextualize any changes in the odds of infection after full vaccination, it is important to understand how they compare to the odds during a period before the onset of vaccine-mediated protection. Thus, we modified the CLR to assess the odds of symptomatic infection over time starting 4 days after the first dose (“post-first dose CLR”), a reference point intended to approximate the unvaccinated state (see Methods). Of 143,443 individuals who received at least one dose of mRNA-1273 with no positive SARS-CoV-2 tests prior to vaccination, 22,864 underwent symptomatic testing after their first dose (Table S4, Supplementary Material).

With the post-first dose CLR stratified by the calendar date of testing, there were 2,684 cases and 17,691 controls contributing to analyzable strata (Tables S4–S5, Supplementary Material). The adjusted odds of symptomatic infection decreased from 4 days after the first dose through the expected second dose and full vaccination dates (e.g. OR_Day 10_: 0.71, 95% CI: 0.42–1.21; OR_Day 28_: 0.15, 95% CI: 0.09–0.25; OR_Day 42_: 0.05, 95% CI: 0.03–0.09), corresponding to the onset of vaccine effectiveness (Figure 2B and Table 1). Increasing odds at later time points indicated a reduction in protection against symptomatic infection compared to the expected full vaccination date (e.g. OR_Day 192_: 0.16; 95% CI: 0.11–0.24; OR_Day 242_: 0.20; 95% CI: 0.14–0.30; OR_Day 292_: 0.26; 95% CI: 0.17–0.39; Figure 2B and Table 1). A similar pattern was observed among individuals who were at least 65 years old (Figure S3A and Table S6, Supplementary Material).

With the post-first dose CLR stratified by the calendar date of vaccination, there were 2,774 cases and 24,864 controls in analyzable strata (Tables S4 and S7, Supplementary Material). Again, the adjusted odds of symptomatic infection decreased through the date of full vaccination (e.g. OR_Day 10_: 0.60, 95% CI: 0.36–0.98; OR_Day 28_: 0.10, 95% CI: 0.06–0.17; OR_Day 42_: 0.05, 95% CI: 0.03–0.09; Figure S2B, Supplementary Material; Table 1). The odds were higher at later time points, indicating a reduction in protection against symptomatic infection over time (e.g. OR_Day 192_: 0.39; 95% CI: 0.25–0.60; OR_Day 242_: 0.58; 95% CI: 0.38–0.90; OR_Day 292_: 0.99; 95% CI: 0.63–1.55; Figure 2B and Table 1). A similar trend was observed among individuals who were at least 65 years old (Figure S3B and Table S6, Supplementary Material).

## Discussion

Taken together, these data suggest that the strong protection against symptomatic SARS-CoV-2 infection which is conferred by vaccination with mRNA-1273 wanes over time. This trend is consistent with updated results from the phase 3 clinical trial of mRNA-1273, in which it was found that that individuals vaccinated by October 2020 (median follow-up: 13 months) were less protected against breakthrough infection during July and August 2021 than those vaccinated after December 1 2020 (median follow-up: 8 months) (33). This is also consistent with a recent large real-world analysis of a veteran population in the United States, which demonstrated waning effectiveness of mRNA-1273 over time through November 2021 (34). The current study provides additional value by analyzing a longer period which extends through the era of the Omicron variant, which marked the largest surge to date in the United States among both vaccinated and unvaccinated individuals. Given that approximately 100 million Americans have now received booster doses, we highlight the importance of continuing to monitor the durability of protection afforded by both primary and booster vaccination with mRNA-1273.

Both approaches that we considered—namely, stratifying the CLR on the date of testing or the date of vaccination—indicate that the effectiveness of mRNA-1273 against symptomatic infection wanes over time. However, the point estimates were quite different between these approaches, with stratification by vaccination date suggesting considerably stronger waning. It is likely that the true trend lies between their estimates. Unfortunately, because our primary variable of interest itself is mathematically defined by these two dates (i.e. Time Since Vaccination = Date of Testing–Date of Vaccination), it is not feasible to control for both of these variables in a single method. It is thus important to recognize the strengths and weaknesses of both approaches.

Stratification on the time of testing controls for community level exposure, NPI practices in place at the time of symptom onset, and (perhaps most importantly) the current local variant landscape, but it ignores differences in the underlying characteristics of individuals vaccinated during different phases of the rollout. Such characteristics certainly include age, comorbidities, and occupation, and may include other factors such as willingness to be vaccinated, likelihood of interaction with the healthcare system, and the symptomaticity threshold required to seek out a COVID-19 test. While we do adjust for age and several COVID-19 associated comorbidities by including them as covariates in the model, we are not able to control for these other characteristics. On the other hand, stratification on the time of vaccination intentionally controls for these characteristics, but makes it more difficult to account for the effects of variants and NPIs. We do try to address this shortcoming by adding the dominant variant and county-level COVID-19 incidence as covariates to the model. However, because Omicron is both the latest and most immunoevasive variant to date, it is possible that this method would not adequately discriminate between the established reduction in vaccine effectiveness against Omicron and a natural decline in vaccine effectiveness over time (35–41). That said, the more pronounced waning signal suggested by this approach is still quite relevant, as it provides an alternative view of “real world waning” that occurs as a function of both time and the introduction of newly evolved SARS-CoV-2 variants.

In addition to assessing waning protection after full vaccination, it is important to contextualize this analysis by estimating protection compared to the unvaccinated state. Because the study cohort only included vaccinated individuals, it was not possible to establish this true baseline rate of symptomatic infection at any given time in the absence of vaccination. However, to approximate this, we leveraged the fact that the 1 to 2 weeks after the first mRNA vaccine dose can serve as a proxy for the unprotected state (3, 4). The odds of symptomatic infection at the expected date of full vaccination (42 days after the first dose) compared to this unprotected state were 0.05 when the CLR was stratified on the date of testing or the date of vaccination, respectively. This corresponds to an estimated effectiveness of about 95% by both methods, consistent with the expected protection based on results of the phase 3 randomized trial and several real world effectiveness studies (3, 5, 9, 12, 13, 42). By about 240 days after the first dose, these relative odds increased to 0.20 and 0.58 when stratifying on the date of testing or vaccination, respectively, suggesting a decline in effectiveness to approximately 40% to 80% over the 200 days following full vaccination. This estimate is quite consistent with the previously reported decline in mRNA-1273 effectiveness from 89% to 58% in a large United States veteran population over a similar period (34).

This study has limitations. First, the cohort is over 90% Caucasian and, thus does not reflect the broader population of vaccinated individuals in the United States or globally. Second, there are individual-level SARS-CoV-2 exposure risks that could not be directly accounted for in our analyses, such as adherence to recommendations regarding masking, travel, and social distancing. As a retrospective observational study, there also may be additional unconsidered confounding factors that were unequally distributed within the study population. Third, it is challenging to select and interpret an appropriate negative control outcome. Ideally, such an outcome would be related to the symptomatic presentation to the clinic but unaffected by vaccination status (or time elapsed since vaccination). For example, prior studies of influenza vaccine effectiveness have considered the lab test-based diagnosis of another respiratory infection as a negative control outcome (43). However, the impact of the COVID-19 pandemic on the incidence and diagnosis of other respiratory illnesses (e.g. low rates of influenza until the end of 2021) made it difficult to consider such outcomes as robust negative controls (44–46). Fourth, because we censored individuals after receiving a third dose of mRNA-1723 (if applicable), any increase in effectiveness or durability attributable to booster doses is not reflected in this study. Finally, due to the low absolute number of COVID-19 associated hospital admissions, ICU admissions, and deaths after full vaccination with mRNA-1273 in this study, there was not adequate power to assess vaccine durability against severe illness.

mRNA-1273 demonstrated strong protection against symptomatic and severe disease in clinical trials and the real world setting during early phases of the vaccine rollout internationally (3, 5, 9,13, 27, 42). This study suggests that mRNA-1273 strongly protects against symptomatic infection for at least 8 months after full vaccination, but its effectiveness declines over time. Moreover, the reduced effectiveness at later time points was amplified by the introduction of the highly immunoevasive Omicron variant. It is important to continue delivering first and second vaccine doses to as many people as possible, while also continuing to administer boosters to eligible and at-risk populations. Further, it will be important to continuously evaluate the durability of protection afforded by mRNA-1273 and other vaccines both over time and in the context of any new SARS-CoV-2 variants following Omicron.

## Materials and Methods

### Study population

This is a retrospective study of individuals who were vaccinated with mRNA-1273 between 2020 December 15 and 2022 January 27, and who subsequently underwent PCR testing for suspected symptomatic SARS-CoV-2 infection at the Mayo Clinic. According to the CDC, full vaccination with mRNA-1273 is defined as beginning 14 days after the second dose (47). This study was reviewed and deemed exempt by the Mayo Clinic institutional review board. Those who had specifically opted out of inclusion of electronic medical records in research were excluded. Inclusion and exclusion criteria were defined as follows:

#### Inclusion criteria

1. Age greater than or equal to 18 years as of 2020 December 15.
2. Received two doses of mRNA-1273 per-protocol, with the first dose administered on or after 2020 December 15. Per-protocol mRNA-1273 vaccination was defined as two doses administered 25 to 38 days apart with no doses of other COVID-19 vaccines (i.e. BNT162b2 and Ad26.COV2.S) administered at any time before the second dose.
3. At least one clinical encounter at the Mayo Clinic in the 3 years preceding the study start date (i.e. between 2017 December 15 and 2020 December 15), per the electronic health record.

#### Exclusion criteria

1. Any positive SARS-CoV-2 PCR test prior to the date of full vaccination.

The derivation of this study population is illustrated in Figure S1 (Supplementary Material), and the demographic and clinical characteristics of the cohort along with the underlying fully vaccinated population is shown in Table 1.

### Study design

We performed a test-negative case-control analysis to assess whether the protection conferred by mRNA-1273 wanes over time, similar to a study design described previously to analyze intraseason waning effectiveness of influenza vaccination (43). To do so, we used CLR to assess the odds of symptomatic SARS-CoV-2 infection and two negative control outcomes (non-COVID-19 hospitalization and non-COVID-19 pneumonia) over time after full vaccination, while adjusting for relevant covariates. Symptomatic infection was defined as a positive result from a SARS-CoV-2 PCR test that was not designated as “asymptomatic” by the ordering provider (subsequently referred to as “symptomatic tests”). Because we expect the date of full vaccination to approximate the time of maximal protection, we assess vaccine durability by estimating the odds of symptomatic infection at 50, 100, 150, 200, and 250 days after this time point.

### Definitions of cases, controls, and at-risk time

Cases were defined as the first positive symptomatic test for a given individual; if an individual contributed multiple positive tests, only their first test was included as a case. Controls were defined as negative symptomatic tests, which did not occur after any prior positive SARS-CoV-2 PCR tests (asymptomatic or symptomatic). Individuals who met the inclusion and exclusion criteria outlined above were eligible to contribute cases and controls from their date of full vaccination until they (i) had any positive test result (symptomatic or asymptomatic), (ii) received a third dose of any COVID-19 vaccine (mRNA-1273, BNT162b2, or Ad26.COV2.S), (iii) died, or (iv) reached the end of the study period. If an individual contributed a negative symptomatic test 15 or fewer days before a positive test, that negative test was excluded as a possible false negative. If an individual contributed multiple negative symptomatic tests within 15 days of each other, then one of those tests was randomly selected as a control while the others were dropped; this step was taken to avoid counting multiple controls from a potential single symptomatic illness. Further, if an individual contributed more than three negative symptomatic tests over the study duration, then three tests were randomly selected as controls while the others were dropped, as was recently described in a test-negative case-control study of COVID-19 vaccine effectiveness (8).

As a negative control analysis, we assessed protection against non-COVID-19 hospitalization and non-COVID-19 pneumonia, outcomes which we do not expect to be impacted by time since vaccination. In other test-negative designs of influenza vaccine effectiveness, the diagnosis of other respiratory infections was considered as the negative control (43). Because of the myriad impacts of the pandemic and NPIs on other respiratory infections (e.g. very low rates of influenza in 2020 and through most of 2021), such an approach may not be valid here (48). Although non-COVID-19 related hospitalization could be impacted by factors such as changes in healthcare-seeking behavior (including elective procedures) after vaccination, it appeared to be a reasonable negative control to evaluate. For non-COVID-19 hospitalization, cases were defined as instances in which an individual experienced a negative symptomatic test (i.e. ruled out for COVID-19 diagnosis) and was subsequently admitted to the hospital within 14 days. Controls were defined as instances in which an individual experienced a negative symptomatic test and was not subsequently admitted to the hospital within 14 days. Individuals who met the inclusion and exclusion criteria outlined above were eligible to contribute cases and controls from their date of full vaccination until they (i) were hospitalized within 14 days of a negative symptomatic test, (ii) received a third dose of any COVID-19 vaccine (mRNA-1273, BNT162b2, or Ad26.COV2.S), (iii) died, or (iv) reached the end of the study period. The same rules were applied as described above for cases in which an individual contributed (i) a negative test shortly before contributing a positive test, (ii) multiple negative symptomatic tests within 15 days of each other, or (iii) more than three negative symptomatic tests over the duration of the study. Because 14 days of follow-up were required after a positive symptomatic test to observe this outcome, cases and controls were only considered from tests that were performed on or before 2022 January 17 (14 days before the last date of data collection). A similar process was followed for non-COVID-19 pneumonia, except that cases were defined by the presence of at least one corresponding ICD-10 code (J12-J18, with the exceptions of J12.81, J12.82, and J12.89) within 14 days of a negative symptomatic test.

### Primary exposure, covariates, and stratification factors

Variables that are potentially associated with the likelihood of eligibility for vaccination at a given time, seeking out vaccination, testing positive for SARS-CoV-2, or experiencing severe COVID-19 were included as covariates or stratification variables in the regression models. The primary exposure of interest and each such other variable, denoted as X_1_ to X_15_ in the regression equation listed in the *Statistical Analysis* section, is described below.

#### Primary exposure

- X_1_: time since full vaccination, defined as the number of days between the symptomatic PCR test and the date of full vaccination. This variable was modeled as a linear spline with knots at 50-day intervals since the full vaccination date. As described above, the date of full vaccination is expected to correspond to a time of maximal protection and, thus was considered as the reference. Results are presented as the odds of symptomatic infection at each knot relative to this reference.

#### Covariates

- X_2_: age in years as of the study start date (2020 December 15), modeled as a linear spline with knots at 25, 35, 45, 55, 65, 75, and 85 years. The minimum age (18 years old) was considered the reference. Results are presented as the odds of symptomatic infection at each subsequent knot relative to this reference.
- X_3_ to X_10_: individual comorbidity categories, binarized based on whether the individual had at least one instance of a corresponding ICD-9 or ICD-10 code in the 5 years prior to the study period. The comorbidity categories included cardiovascular disease, pulmonary disease, diabetes, kidney disease, liver disease, HIV/AIDS, cancer, and obesity.
- X_11_: race, categorized into seven groups (listed alphabetically: Asian, Black/African American, Native American, Native Hawaiian/Pacific Islander, other, White, and unknown). White was considered the reference category because it comprised the majority of individuals in the study.
- X_12_: ethnicity, categorized into three groups (listed alphabetically: Hispanic/Latino, not Hispanic/Latino, and unknown). “Not Hispanic/Latino” was considered the reference category because it comprised the majority of individuals in the study.
- X_13_: sex, categorized into three groups (listed alphabetically: female, male, and unknown). Female was considered the reference category.

#### Stratification factors

- X_14_: county of residence at the time of testing for the individual who underwent the symptomatic test.
- X_15_: calendar time of test, categorized in 1-week intervals starting on the date of the first symptomatic test after full vaccination.

### Determination of comorbidities

We used the *comorbidity* package (version 0.5.3) in R (version 4.1.0, www.r-project.org, Vienna, Austria) to identify ICD-9 and ICD-10 codes that correspond to the comorbidity categories listed above. For each individual, we extracted all such diagnosis codes in the Mayo Clinic electronic health record from the 5 years preceding this study (i.e. between 2015 December 15 and 2020 December 15). The eight comorbidity categories were defined as one or more diseases from the Elixhauser score as follows:

1. Cardiovascular disease: congestive heart failure (chf), cardiac arrhythmias (carit), valvular disease (valv), pulmonary circulation disorders (pcd), peripheral vascular disorders (pvd), uncomplicated hypertension (hypunc), and complicated hypertension (hypc).
2. Pulmonary disease: chronic pulmonary disease (cpd).
3. Diabetes: uncomplicated diabetes (diabunc) and complicated diabetes (diabc).
4. Kidney disease: renal failure (rf).
5. Liver disease: liver disease (lf).
6. HIV/AIDS: AIDS/HIV (aids).
7. Cancer: lymphoma (lymph), metastatic cancer (metacanc), and solid tumor without metastasis (solidtum).
8. Obesity: obesity (obes).

### Statistical analysis

Briefly, for each outcome (i.e. symptomatic SARS-CoV-2 infection, non-COVID-19 hospitalization, and non-COVID-19 pneumonia), we fit a CLR model to estimate the odds of experiencing the outcome of interest each day after the date of full vaccination compared to the odds of experiencing that outcome on the date of full vaccination, while adjusting for the covariates described above.

The CLR models were each defined by the equation

$log( {\frac{{{p_{Outcome}}}}{{1\ - \ {p_{Outcome}}}}} )\ = $ β_0_ + β_1_X_1_ + β_2_X_2_ + . . . + β_13_X_13_ + Strata[X_14_, X_15_], where the covariates and conditioning variables X_1_ to X_15_ are described in the section above.

Models were fit using the *clogit* function from the *survival* package (version 3.2.11) in R (Version, 4.1.0, www.r-project.org, Vienna, Austria). Confidence intervals and tests for individual covariates were based upon the Wald method, and the Efron method was used to approximate the conditional likelihood. Odds ratios were considered statistically significant if the confidence interval did not include 1. In addition, Nagelkerke *R*-squared values are reported for each model in Table S8 (Supplementary Material).

### Secondary analysis: odds of infection relative to the first vaccine dose rather than full vaccination

The primary analysis estimates the change in odds of infection over time relative to the time of maximal vaccine protection without providing the important context of the baseline risk of infection in the absence of vaccination. In our study population, which consists entirely of vaccinated individuals with no prior history of COVID-19 diagnosis, it is not possible to directly measure this risk of infection in the unvaccinated state. However, the protective effect of mRNA-1273 sets in after approximately 2 weeks, and we reasoned that the risk of infection in the unvaccinated state could be approximated by the risk of infection shortly after initial vaccination (e.g. within Days 4 to 10 after the first dose) (3, 5). We, thus modified the inclusion and exclusion criteria from our primary analysis as follows.

#### Inclusion criteria

1. Age greater than or equal to 18 years as of 2020 December 15.
2. Received at least one dose of mRNA-1273 on or after 2020 December 15.
3. At least one clinical encounter at the Mayo Clinic in the 3 years preceding the study start date (i.e. between 2017 December 15 and 2020 December 15), per the electronic health record.

#### Exclusion criteria

1. Any positive SARS-CoV-2 PCR test prior to the first dose of mRNA-1273 or in the 3 days after this first dose.
2. Received one or more doses of another COVID-19 vaccine (BNT162b2 or Ad26.COV2.S) on or before 2020 December 15.

Cases and controls were defined as described above but with modifications to the censoring protocol. Specifically, individuals who met the inclusion and exclusion criteria were eligible to contribute cases and controls from 4 days after their first vaccine dose until they (i) had any positive test result (symptomatic or asymptomatic), (ii) went off-protocol for their vaccination regimen (i.e. received a second dose of mRNA-1273 less than 25 days after the first dose, did not receive a second dose of mRNA-1273 by 35 days after their first dose, or received a dose of a different COVID-19 vaccine within 35 days of their first dose), (iii) received a third dose of any COVID-19 vaccine, (iv) died, or (v) reached the end of the study period. Note that at-risk time was defined to begin 4 days after the first dose (rather than the first day after the first dose) for two reasons: (i) individuals with respiratory symptoms were often encouraged to delay vaccination, resulting in a potential bias toward lower symptomatic infection rates immediately following vaccination; and (ii) individuals who develop symptomatic COVID-19 shortly after vaccination may attribute their symptoms to vaccine side effects, resulting in a likely delay of testing.

The same CLR model described above for the main analysis was applied, except that the “Time since vaccination” variable was now modeled as a linear spline with the following knots: 10, 14, 28, 42, 92, 142, 192, 242, 292, and 342 days after the first dose. It is recommended that the second dose of mRNA-1273 is administered 28 days after the first, with full vaccination thus expected to start 42 days after the first dose. The spline knots after Day 42 were defined as 50-day intervals after this expected date of full vaccination so that they would approximately match the spline knots considered in the primary analysis (i.e. 50-day intervals after the actual date of full vaccination). Because the vaccine is not expected to provide protection until about 2 weeks after the first dose, we considered 4 days after the first dose as a reference time point to approximate unvaccinated status (3, 5). Results are presented as the odds of symptomatic infection at each knot relative to this reference.

### Sensitivity analyses

By performing CLR with stratification by the time of testing, individuals with more time since vaccination in any given stratum will inherently have been vaccinated at earlier times during the vaccine rollout. Because the time of vaccination is likely associated with the risk of SARS-CoV-2 infection and the likelihood of engagement with the healthcare system, this approach could yield biased estimates of the infection odds over time after vaccination. We thus performed a sensitivity analysis in which the CLR was stratified by county, date of full vaccination (in 2-week calendar intervals), and county-level COVID-19 incidence at the time of testing, rather than on county and time of testing (as was the case in the previous model). Here, we also included an additional covariate to capture the dominant SARS-CoV-2 variant at the time of the test; this was not included in our primary model because the variant prevalence was implicitly captured by stratifying on the time of testing. Specifically, the additional variables considered here were:

1. X_16_: dominant SARS-CoV-2 variant, categorized as Alpha, Delta, Omicron, neither, or unknown. The prevalence of the Alpha, Delta, and Omicron variants was determined for each state in twice-monthly intervals (i.e. from the first to the 15th day of each month, and from the 16th to the last day of each month) using publicly deposited whole genome sequences in the NCBI database (49). For a given test, which is characterized by a specific combination of state and twice-monthly interval, this variable was denoted as (i) Alpha if the prevalence of Alpha variant sequences was > 0.5, (ii) Delta if the prevalence of Delta variant sequences was > 0.5, (iii) Omicron if the prevalence of the Omicron variant sequences was > 0.5, (iv) neither if the prevalence of Alpha, Delta, and Omicron variant sequences was < 0.5, or (iv) unknown if there were fewer than 50 sequences deposited.
2. X_17_: community level exposure risk, modeled as a linear spline with knots at 10, 25, 50, 75, 100, and 150 cases per 100,000 individuals. For a given county on a given day of testing, community exposure risk was proxied by the trailing 7-day average COVID-19 incidence (50).
3. X_18_: community level exposure risk, categorized into seven buckets based on the same values used for the spline knots in variable X_17_.
4. X_19_: calendar time of full vaccination for the individual who underwent the symptomatic test, categorized in 2-week intervals.

The CLR model was then defined by the equation

$log( {\frac{{{p_{Outcome}}}}{{1\ - \ {p_{Outcome}}}}} )\ = $ β_0_ + β_1_X_1_ + β_2_X_2_ + . . . + β_13_X_13_ + β_16_X_16_ + β_17_X_17_ + Strata[X_14_, X_18_, X_19_], where the covariates and conditioning variables X_1_ to X_13_ are the same as described above for the primary analysis. This equation mirrors that used in the primary analysis, except that X_16_ (dominant SARS-CoV-2 variant) and X_17_ (splined community level exposure risk) were added as covariates, and the stratifying variable X_15_ (calendar time of test) was replaced with X_18_ (bucketed community level exposure risk) and X_19_ (calendar time of vaccination).

### Multicollinearity analysis

To assess multicollinearity among the covariates for each CLR model, we computed the Variance Inflation Factor (VIF) for each of the nonstrata covariates in each model. These values are provided in Table S9 (Supplementary Material). A cutoff VIF threshold of 5 was used to identify covariates which are significantly collinear with respect to the other model covariates. VIF values were computed using the *mctest* package (version 1.3.1) in R.

## Supplementary Material

pgac058_Supplemental_File

## Data Availability

The datasets supporting the current study have not been deposited because they contain personally identifiable information from human subjects. This data may be made available from the corresponding author on request (Venky Soundararajan; venky@nference.net). A proposal with a detailed description of the study objectives and statistical analysis plan will be needed to evaluate the reasonability of requests. Deidentified data will be provided after approval from the lead contact and the Mayo Clinic's standard IRB process for such requests.

## Notes

**Competing Interest:** A.P., P.J.L., A.V., C.P. and V.S. are employees of nference. nference is collaborating, or has collaborated, with Moderna, Janssen, Pfizer, and other biopharmaceutical companies on data science initiatives unrelated to this study. These collaborations had no role in study design, data collection and analysis, decision to publish, or preparation of the manuscript. J.C.O. has received small grants from nference, Inc., and personal consulting fees from Bates College and Elsevier Inc. All of these activities are outside of the present work. M.D.S. received research funding for the HEROES Together vaccine SE registry from Pfizer via Duke University. A.V. reports being an inventor for Mayo Clinic Travel App interaction with Smart Medical Kit and Medical Kit for Pilgrims. A.D.B. is supported by grants from the NIAID (grants AI110173 and AI120698), Amfar (#109593), and Mayo Clinic (HH Shieck Khalifa Bib Zayed Al-Nahyan Named Professorship of Infectious Diseases). A.D.B. is a paid consultant for Abbvie, Gilead, Freedom Tunnel, Pinetree therapeutics Primmune, Immunome, MarPam, and Flambeau Diagnostics, is a paid member of the DSMB for Corvus Pharmaceuticals, Equilium, and Excision Biotherapeutics, has received fees for speaking for Reach MD and Medscape, owns equity for scientific advisory work in Zentalis and nference, and is the founder and President of Splissen therapeutics. W.K., J.E.G., H.L.G., L.B., D.W.C., and L.L.S. have no interests to disclose. Mayo Clinic holds equity in the company sponsoring the analysis presented in this manuscript.
