# Supplementary material for: Durability analysis of the highly effective mRNA-1273 vaccine against COVID-19
Source: PNAS Nexus. 2022 May 20;1(2):pgac058. doi: 10.1093/pnasnexus/pgac058 (PMC9802296; doi:10.1093/pnasnexus/pgac058)
Supplement: pgac058_Supplemental_File [file pgac058_supplemental_file.pdf]

Supplementary Data

Supplemental Figures

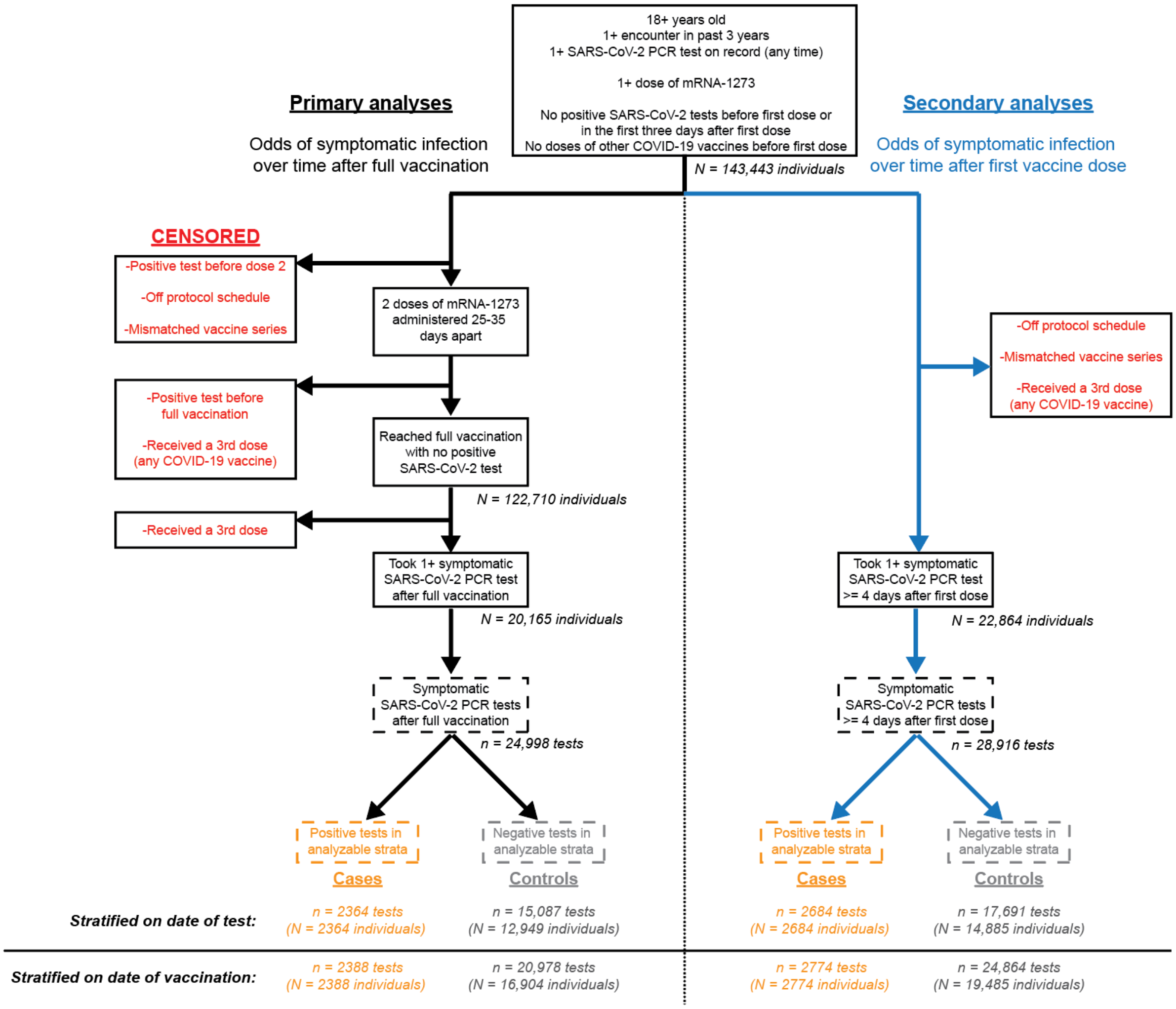

Figure S1. Cohort development flowchart showing derivation of the various cohorts analyzed in this study. Cohort derivation is depicted for four different study designs that were considered. On the left, the flow chart depicts the cohort derivation to analyze the odds of symptomatic infection after full vaccination, stratified by either the date of testing or the date of vaccination. On the right, the flow chart depicts the cohort derivation to analyze the odds of symptomatic infection after the first dose, again stratified by either the date of testing or the date of vaccination. For the conditional logistic regression, a “stratum” is defined as a unique combination of county (geography) and calendar time (of either testing or vaccination). An “analyzable stratum” is any such stratum which contains at least one case and at least one control. An individual was considered to have gone “off protocol schedule” if they (i) received a second dose of mRNA-1273 less than 25 days after the first dose, (ii) did not receive a second dose of mRNA-1273 by 35 days after the first dose, or (iii) received a dose of a different COVID-19 vaccine within 35 days of the first mRNA-1273 dose. Boxes with solid outlines correspond to counts of individuals, and boxes with dotted outlines correspond to counts of tests. In the test-negative analyses, cases and controls were defined at the level of symptomatic tests. One individual is allowed to contribute multiple negative symptomatic tests, provided that those tests were separated from each other by at least 15 days. If an individual experienced any negative symptomatic tests prior to a positive test, those negative tests can contribute to the control set and the positive test can contribute to the case set.

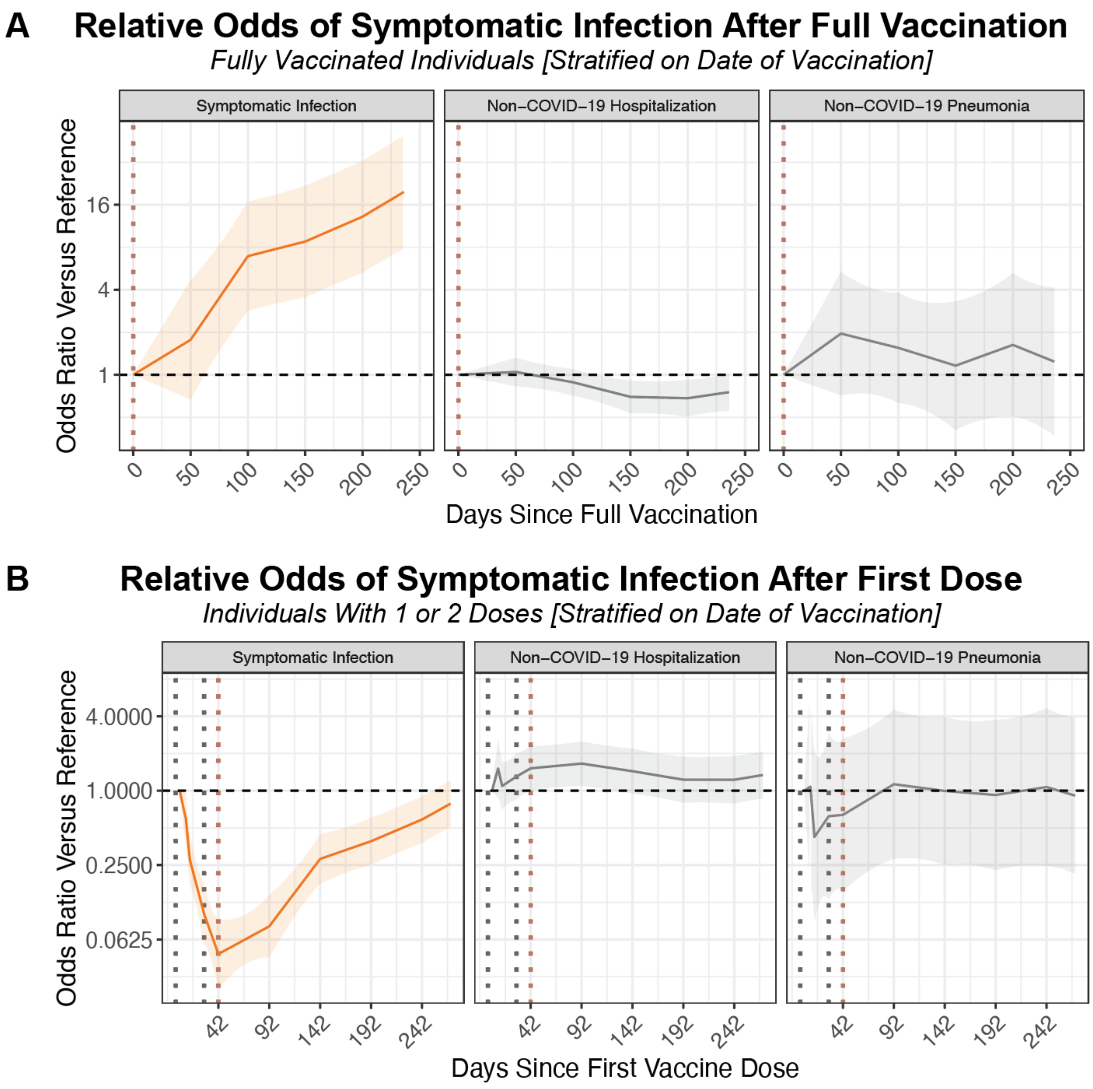

Figure S2. Relationship between time since vaccination and the adjusted odds of experiencing each outcome of interest in the secondary analysis, with stratification by the date of vaccination rather than the date of testing. (A) Adjusted odds ratios of outcomes over time relative to the date of full vaccination, defined as 14 days after the second dose of mRNA-1273. Odds ratios correspond to the odds of experiencing the outcome at a given time *t* divided by the odds of experiencing that outcome at the reference date (*t* = 0 days since full vaccination*)*, adjusted for several demographic and clinical covariates via conditional logistic regression (CLR). (B) Adjusted odds ratios of outcomes over time relative to four days after the first mRNA-1273 dose, which is intended to approximate the unvaccinated status. Odds ratios correspond to the odds of experiencing the outcome at a given time *t* divided by the odds of experiencing that outcome at the reference date (*t* = 4 days since first vaccine dose*)*, again adjusted for demographic and clinical covariates via CLR. The outcomes are symptomatic SARS-CoV-2 infection (shown in orange), non-COVID-19 associated hospitalization, and non-COVID-19 pneumonia (negative control outcomes shown in gray). For both panels, the CLR was stratified on residential county, the date of full vaccination, and the 7-day trailing county-level COVID-19 incidence on the date of the SARS-CoV-2 PCR test.

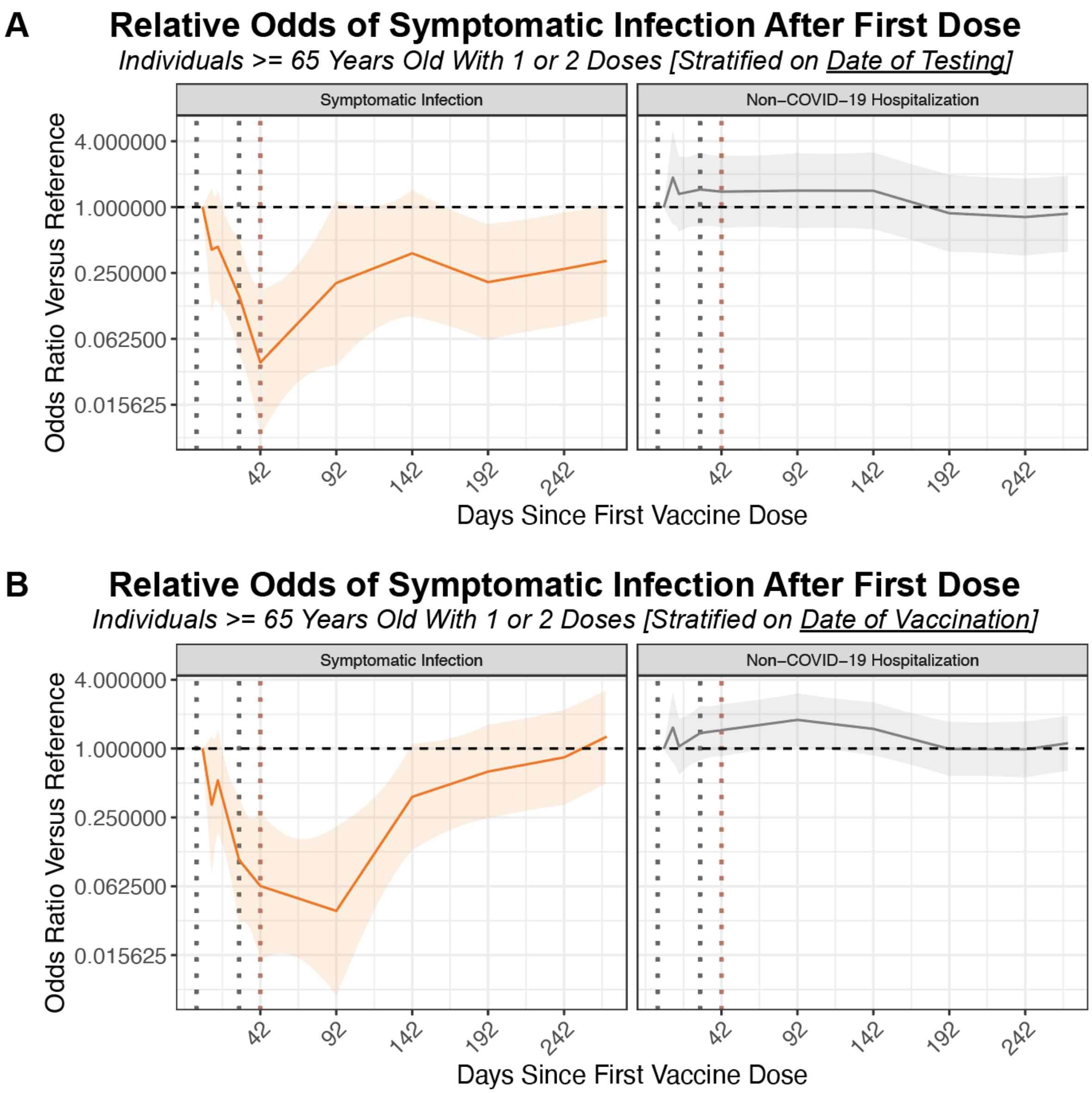

Figure S3. Relationship between time since first vaccine dose and the adjusted odds of experiencing each outcome of interest in individuals at least 65 years old. Adjusted odds ratios of outcomes over time relative to four days after the first mRNA-1273 dose, which is intended to approximate the unvaccinated status. Odds ratios correspond to the odds of experiencing the outcome at a given time *t* divided by the odds of experiencing that outcome at the reference date (*t* = 4 days since first vaccine dose), adjusted for demographic and clinical covariates via CLR. The outcomes are symptomatic SARS-CoV-2 infection (shown in orange) and non-COVID-19 associated hospitalization (negative control outcome shown in gray). (A) The CLR was stratified on residential county and the date of SARS-CoV-2 PCR testing. (B) The CLR was stratified on residential county, the date of full vaccination, and the 7-day trailing county-level COVID-19 incidence on the date of the SARS-CoV-2 PCR test.

Supplemental Tables

Table S1. Demographic and clinical characteristics of cases and controls for primary analysis of symptomatic infection after full vaccination with mRNA-1273. The underlying population corresponds to the set of individuals who received their first mRNA-1273 dose on or after December 15, 2020 and were fully vaccinated per protocol (i.e. with two doses administered 25-35 days apart and with no prior positive SARS-CoV-2 PCR tests before the date of full vaccination). The eligible population corresponds to the subset of the underlying population who underwent at least one symptomatic test after the date of full vaccination. Cases correspond to the first positive symptomatic test for a given individual in the eligible population; by definition, the number of individuals contributing cases is the same as the number of cases. Controls correspond to negative symptomatic tests after full vaccination which occurred before the given individual has experienced any positive SARS-CoV-2 PCR tests; an individual can contribute multiple controls during the study period, so the number of individuals in the control population is less than the total number of tests (controls) contributed. Because an individual can contribute negative tests (controls) prior to contributing a positive test, the number of individuals in the eligible population is smaller than the sum of the number of individuals in the case and control populations. Sub-sampling in the control population refers to the process in which negative tests from a given individual were (i) excluded if they occurred after a positive test or within the 15 days before a positive test (possible false negative), (ii) randomly sampled if they occurred within 15 days of each other (possibly during the same symptomatic illness), and (iii) randomly sampled if the individual contributed more than three negative tests during the study period. A stratum (defined by the regression equation as a unique combination of county and calendar week of testing) is considered analyzable if it includes at least one case and at least one control, because strata including only cases or only controls do not contribute to the estimation of the regression coefficients. For all cases and controls, all summarized characteristics correspond to only individuals who contributed at least one symptomatic test to an analyzable stratum.

**Characteristic**			**Stratified on Date of PCR Test**		**Stratified on Date of Vaccination**	
	**Underlying Population**<br><br>Fully vaccinated per-protocol	**Eligible Population**<br><br>Fully vaccinated per-protocol with subsequent symptomatic test	**Case Population**<br><br>1+ positive symptomatic test after full vaccination	**Control Population**<br><br>1+ negative symptomatic test after full vaccination, sub-sampled	**Case Population**<br><br>1+ positive symptomatic test after full vaccination	**Control Population**<br><br>1+ negative symptomatic test after full vaccination, sub-sampled
Number of individuals	122710	20165	2364	12949	2388	16904
Number of symptomatic tests						
Total		24998	2364	15087	2388	20978
Bucketed by time since full vaccination date						
- Days 0-29			21	324	18	1785
- Days 30-59			28	350	25	1588
- Days 60-89			58	643	53	1788
- Days 90-119			147	1495	149	2284
- Days 120-149			270	2438	274	2918
- Days 150-179			478	3434	483	3714
- Days 180-209			508	3112	514	3297
- Days 210-239			449	1913	458	2073

- Days 240-269<br>- Days 270-299<br>- Days 300+			261<br>103<br>41	878<br>380<br>120	266<br>106<br>42	988<br>420<br>123
Age (years)						
- Mean (sd)	57.6 (18.0)	55.8 (18.4)	52.5 (17.1)	53.9 (18.3)	52.6 (17.1)	56.2 (18.4)
- 18-24	6,776 (5.5%)	1,215 (6.0%)	131 (5.5%)	880 (6.8%)	133 (5.6%)	989 (5.9%)
- 25-34	10,858 (8.8%)	2,178 (10.8%)	288 (12.2%)	1,591 (12.3%)	286 (12.0%)	1,791 (10.6%)
- 35-44	13,784 (11.2%)	2,747 (13.6%)	450 (19.0%)	1,951 (15.1%)	455 (19.1%)	2,254 (13.3%)
- 45-54	15,904 (13.0%)	2,786 (13.8%)	396 (16.8%)	1,897 (14.6%)	400 (16.8%)	2,299 (13.6%)
- 55-64	24,653 (20.1%)	3,842 (19.1%)	448 (19.0%)	2,476 (19.1%)	450 (18.8%)	3,221 (19.1%)
- 65-74	31,673 (25.8%)	4,550 (22.6%)	437 (18.5%)	2,651 (20.5%)	447 (18.7%)	3,906 (23.1%)
- 75-84	13,953 (11.4%)	1,918 (9.5%)	161 (6.8%)	1,006 (7.8%)	163 (6.8%)	1,637 (9.7%)
- 85+	4,805 (3.9%)	929 (4.6%)	53 (2.2%)	497 (3.8%)	54 (2.3%)	807 (4.8%)
State of Residence						
- Arizona	9,755 (7.9%)	865 (4.3%)	93 (3.9%)	562 (4.3%)	91 (3.8%)	718 (4.2%)
- Florida	23,902 (19.5%)	3,024 (15.0%)	294 (12.4%)	1,945 (15.0%)	301 (12.6%)	2,650 (15.7%)
- Iowa	3,099 (2.5%)	177 (0.9%)	9 (0.4%)	15 (0.1%)	15 (0.6%)	54 (0.3%)
- Minnesota	61,277 (49.9%)	11,129 (55.2%)	1,436 (60.7%)	7,367 (56.9%)	1,431 (59.9%)	9,429 (55.8%)
- Wisconsin	17,527 (14.3%)	4,888 (24.2%)	532 (22.5%)	3,060 (23.6%)	547 (22.9%)	4,050 (24.0%)
- Other	7,150 (5.8%)	82 (0.4%)	0 (0.0%)	0 (0.0%)	3 (0.1%)	3 (0.0%)
Sex						
- Female	65,881 (53.7%)	12,018 (59.6%)	1,400 (59.2%)	7,797 (60.2%)	1,411 (59.1%)	10,097 (59.7%)
- Male	56,822 (46.3%)	8,146 (40.4%)	963 (40.7%)	5,151 (39.8%)	976 (40.9%)	6,806 (40.3%)
- Unknown	7 (0.0%)	1 (0.0%)	1 (0.0%)	1 (0.0%)	1 (0.0%)	1 (0.0%)
Race						
- Asian	2,783 (2.3%)	440 (2.2%)	31 (1.3%)	317 (2.4%)	31 (1.3%)	393 (2.3%)
- Black	3,246 (2.6%)	400 (2.0%)	44 (1.9%)	279 (2.2%)	44 (1.8%)	334 (2.0%)
- Native American	489 (0.4%)	78 (0.4%)	8 (0.3%)	55 (0.4%)	8 (0.3%)	67 (0.4%)
- Hawaiian/Pacific	110 (0.1%)	13 (0.1%)	0 (0.0%)	7 (0.1%)	0 (0.0%)	13 (0.1%)
- White	110,232 (89.8%)	18,775 (93.1%)	2,205 (93.3%)	11,979 (92.5%)	2,229 (93.3%)	15,726 (93.0%)
- Other	2,230 (1.8%)	325 (1.6%)	50 (2.1%)	226 (1.7%)	50 (2.1%)	267 (1.6%)
- Unknown	3,620 (3.0%)	134 (0.7%)	26 (1.1%)	86 (0.7%)	26 (1.1%)	104 (0.6%)
Ethnicity: Hispanic/Latino						
- Yes	4,727 (3.9%)	742 (3.7%)	104 (4.4%)	499 (3.9%)	108 (4.5%)	601 (3.6%)
- No	112,471 (91.7%)	19,178 (95.1%)	2,228 (94.2%)	12,288 (94.9%)	2,248 (94.1%)	16,093 (95.2%)
- Unknown	5,512 (4.5%)	245 (1.2%)	32 (1.4%)	162 (1.3%)	32 (1.3%)	210 (1.2%)
Comorbidities						
- Cancer	6,577 (6.4%)	1,208 (6.2%)	98 (4.3%)	693 (5.6%)	96 (4.2%)	1,051 (6.5%)
- Cardiovascular	23,537 (22.8%)	5,346 (27.6%)	502 (22.2%)	3,212 (25.9%)	507 (22.2%)	4,614 (28.4%)
- Diabetes	7,338 (7.1%)	1,813 (9.4%)	181 (8.0%)	1,054 (8.5%)	184 (8.1%)	1,558 (9.6%)
- HIV/AIDS	60 (0.1%)	14 (0.1%)	1 (0.0%)	9 (0.1%)	1 (0.0%)	12 (0.1%)
- Kidney disease	6,213 (6.0%)	1,460 (7.5%)	126 (5.6%)	796 (6.4%)	127 (5.6%)	1,260 (7.8%)
- Liver disease	4,160 (4.0%)	943 (4.9%)	81 (3.6%)	593 (4.8%)	84 (3.7%)	826 (5.1%)
- Obesity	11,911 (11.6%)	3,129 (16.2%)	350 (15.5%)	1,907 (15.4%)	354 (15.5%)	2,673 (16.4%)
- Pulmonary	9,235 (9.0%)	2,497 (12.9%)	238 (10.5%)	1,567 (12.6%)	241 (10.6%)	2,170 (13.4%)
Dates of full vaccination						
- Earliest	1/21/21	2/2/21	2/2/21	2/2/21	2/2/21	2/2/21
- 25th %	3/16/21	3/13/21	3/16/21	3/16/21	3/16/21	3/13/21
- Median	4/8/21	4/6/21	4/9/21	4/9/21	4/9/21	4/2/21
- 75th %	5/4/21	4/30/21	5/5/21	5/4/21	5/5/21	4/28/21
- Latest	1/31/22	12/31/21	12/31/21	12/17/21	12/3/21	12/10/21

Days between full vaccination and test<br>- Minimum<br>- 25th %<br>- Median<br>- 75th %<br>- Maximum		 0<br>94<br>154<br>197<br>328	 0<br>155<br>190<br>226<br>323	 0<br>133<br>169<br>204<br>328	 1<br>156<br>191<br>227<br>323	 0<br>91<br>151<br>193<br>324

Table S2. Adjusted odds of symptomatic SARS-CoV-2 infection, non-COVID-19 hospitalization, and non-COVID-19 pneumonia for all non-strata covariates except time since vaccination in fully vaccinated individuals, derived from the CLR with stratification on the calendar date of testing. In total, there were 2364 positive symptomatic tests, 3385 non-COVID-19 hospitalizations, and 273 cases of non-COVID-19 pneumonia which contributed to analyzable strata. Adjusted odds were estimated with linear spline equations for age or by exponentiating the coefficients derived from conditional logistic regression models fit separately for each outcome.

		Adjusted Odds Ratio (95% CI)		
**Covariate**	**Level/ Category**	**Symptomatic Infection**<br>**[N = 2364 events]**	**Non-COVID-19 Hospitalization**<br>**[N = 3385 events]**	**Non-COVID-19 Pneumonia**<br>**[N = 273 events]**
Age	18	1 (Reference)	1 (Reference)	1 (Reference)
	25	0.87 (0.56-1.35)	1.77 (0.98-3.22)	1.89 (0.17-20.79)
	35	1.43 (1.02-2.01)	1.32 (0.82-2.13)	0.92 (0.13-6.7)
	45	1.47 (1.03-2.09)	1.72 (1.05-2.81)	0.86 (0.11-6.84)
	55	1.22 (0.86-1.73)	3.01 (1.88-4.81)	4.62 (0.69-31)
	65	1.38 (0.97-1.96)	3.49 (2.19-5.57)	3.76 (0.56-25.13)
	75	1.22 (0.84-1.76)	6.65 (4.17-10.61)	6.3 (0.94-42.24)
	85	1.25 (0.81-1.92)	13.52 (8.43-21.69)	11.23 (1.65-76.56)
Comorbidities	Cardiovascular disease	0.805 (0.697, 0.931)	1.13 (1.01, 1.25)	0.647 (0.423, 0.988)
	Pulmonary disease	0.837 (0.716, 0.979)	1.21 (1.09, 1.34)	2.88 (1.96, 4.23)
	Diabetes	1.06 (0.876, 1.28)	1.3 (1.16, 1.46)	1.08 (0.691, 1.68)
	Kidney disease	1.12 (0.904, 1.39)	1.2 (1.07, 1.34)	1.24 (0.801, 1.93)
	Liver disease	0.868 (0.681, 1.11)	1.34 (1.16, 1.54)	1.86 (1.13, 3.06)
	HIV/AIDS	0.97 (0.125, 7.51)	0.679 (0.233, 1.98)	2.32e-07 (0, Inf)
	Cancer	0.956 (0.764, 1.2)	0.981 (0.867, 1.11)	0.753 (0.467, 1.22)

	Obesity	1.13 (0.977, 1.31)	1.03 (0.923, 1.14)	0.798 (0.529, 1.2)
Race	White/Caucasian	1 (Reference)	1 (Reference)	1 (Reference)
	Asian	0.571 (0.398, 0.821)	1.25 (0.956, 1.64)	0.267 (0.0361, 1.97)
	Black/African American	0.933 (0.684, 1.27)	1.34 (1.01, 1.76)	2.49 (1.01, 6.11)
	Native American	0.817 (0.402, 1.66)	0.986 (0.501, 1.94)	1.04 (0.134, 8.06)
	Native Hawaiian / Pacific Islander	1.81e-06 (0, Inf)	0.835 (0.111, 6.27)	1.01e-06 (0, Inf)
	Other	1.19 (0.871, 1.63)	1.12 (0.784, 1.59)	1.68 (0.472, 5.96)
	Unknown	1.4 (0.886, 2.21)	0.903 (0.45, 1.81)	2.95 (0.318, 27.4)
Ethnicity	Not Hispanic or Latino	1 (Reference)	1 (Reference)	1 (Reference)
	Hispanic or Latino	1.06 (0.845, 1.33)	0.943 (0.738, 1.21)	0.277 (0.0635, 1.21)
	Unknown	1.06 (0.706, 1.6)	0.78 (0.496, 1.23)	0.506 (0.0967, 2.65)
Sex	Female	1 (Reference)	1 (Reference)	1 (Reference)
	Male	1.12 (1.02, 1.22)	1.22 (1.13, 1.31)	1.84 (1.41, 2.41)
	Unknown	1.91 (0.188, 19.5)	NA	NA

Table S3. Adjusted odds of symptomatic SARS-CoV-2 infection, non-COVID-19 hospitalization, and non-COVID-19 pneumonia for all non-strata covariates except time since vaccination in fully vaccinated individuals, derived from the CLR with stratification on the calendar date of vaccination. In total, there were 2388 positive symptomatic tests, 3597 non-COVID-19 hospitalizations, and 288 cases of non-COVID-19 pneumonia which contributed to analyzable strata. Adjusted odds were estimated with linear spline equations for age or by exponentiating the coefficients derived from conditional logistic regression models fit separately for each outcome.

		Adjusted Odds Ratio (95% CI)		
**Covariate**	**Level/ Category**	**Symptomatic Infection [N = 2388 events]**	**Non-COVID-19 Hospitalization [N = 3597 events]**	**Non-COVID-19 Pneumonia [N = 288 events]**
Age	18	1 (Reference)	1 (Reference)	1 (Reference)
	25	0.78 (0.49-1.24)	1.95 (1.04-3.64)	1.04 (0.09-11.71)
	35	1.36 (0.95-1.95)	1.34 (0.81-2.23)	0.57 (0.08-4.22)
	45	1.4 (0.96-2.05)	1.76 (1.04-2.97)	0.43 (0.05-3.44)
	55	1.11 (0.76-1.62)	3.06 (1.85-5.07)	2.51 (0.37-16.96)
	65	1.27 (0.87-1.85)	3.68 (2.24-6.06)	2.37 (0.36-15.76)
	75	1.11 (0.75-1.66)	7.59 (4.61-12.5)	3.98 (0.59-26.69)
	85	1.19 (0.75-1.88)	13.79 (8.34-22.8)	6.28 (0.93-42.28)
Comorbidities	Cardiovascular disease	0.765 (0.659, 0.889)	1.13 (1.01, 1.26)	0.708 (0.463, 1.08)
	Pulmonary disease	0.86 (0.732, 1.01)	1.12 (1.02, 1.24)	2.32 (1.59, 3.41)
	Diabetes	1.05 (0.866, 1.28)	1.27 (1.14, 1.42)	1.08 (0.698, 1.68)
	Kidney disease	1.1 (0.877, 1.37)	1.16 (1.03, 1.29)	1.27 (0.827, 1.94)
	Liver disease	0.846 (0.656, 1.09)	1.3 (1.14, 1.5)	1.88 (1.15, 3.09)
	HIV/AIDS	1.57 (0.216, 11.5)	1.05 (0.387, 2.86)	1.1e-06 (0, Inf)
	Cancer	0.984 (0.782, 1.24)	1.02 (0.903, 1.15)	0.835 (0.522, 1.34)

	Obesity	1.18 (1.01, 1.37)	1.07 (0.962, 1.19)	0.842 (0.557, 1.27)
Race	White/Caucasian	1 (Reference)	1 (Reference)	1 (Reference)
	Asian	0.554 (0.379, 0.81)	1.25 (0.952, 1.63)	0.312 (0.0426, 2.28)
	Black/African American	0.899 (0.644, 1.26)	1.34 (1.01, 1.77)	1.6 (0.554, 4.64)
	Native American	0.69 (0.329, 1.45)	0.924 (0.471, 1.81)	1.18 (0.148, 9.45)
	Native Hawaiian / Pacific Islander	2.28e-06 (0, Inf)	0.772 (0.101, 5.93)	2.59e-06 (0, Inf)
	Other	1.21 (0.874, 1.68)	1.1 (0.78, 1.56)	2.58 (0.722, 9.22)
	Unknown	1.55 (0.97, 2.46)	0.75 (0.361, 1.56)	0.707 (0.0363, 13.8)
Ethnicity	Not Hispanic or Latino	1 (Reference)	1 (Reference)	1 (Reference)
	Hispanic or Latino	1.04 (0.829, 1.32)	0.968 (0.757, 1.24)	0.248 (0.0554, 1.11)
	Unknown	1.13 (0.752, 1.71)	0.835 (0.534, 1.31)	0.755 (0.121, 4.71)
Sex	Female	1 (Reference)	1 (Reference)	1 (Reference)
	Male	1.11 (1.01, 1.21)	1.25 (1.16, 1.34)	2.07 (1.58, 2.72)
	Unknown	NA	NA	NA
Dominant Variant	Alpha	1 (Reference)	1 (Reference)	1 (Reference)
	Delta	0.991 (0.501, 1.96)	1.05 (0.856, 1.29)	1.33 (0.606, 2.93)
	Omicron	1.53 (0.736, 3.17)	0.92 (0.597, 1.42)	0.269 (0.048, 1.5)
	Neither	1.61 (0.598, 4.33)	0.878 (0.655, 1.18)	1.1 (0.34, 3.56)
	Unknown	1.32 (0.646, 2.71)	1.38 (1.14, 1.66)	1.11 (0.541, 2.28)

Table S4. Demographic and clinical characteristics of cases and controls for secondary analysis of symptomatic infection after first dose of mRNA-1273. The underlying population corresponds to the set of individuals who received their first mRNA-1273 dose on or after December 15, 2020 and had no positive SARS-CoV-2 PCR tests before four days after their first dose. The eligible population corresponds to the subset of the underlying population who underwent at least one symptomatic test four or more days after their first vaccine dose. Cases correspond to the first positive symptomatic test for a given individual in the eligible population; by definition, the number of individuals contributing cases is the same as the number of cases. Controls correspond to negative symptomatic tests after the first vaccine dose which occurred before the given individual has experienced any positive SARS-CoV-2 PCR tests; an individual can contribute multiple controls during the study period, so the number of individuals in the control population is less than the total number of tests (controls) contributed. Because an individual can contribute negative tests (controls) prior to contributing a positive test, the number of individuals in the eligible population is smaller than the sum of the number of individuals in the case and control populations. Sub-sampling in the control population refers to the process in which negative tests from a given individual were (i) excluded if they occurred after a positive test or within the 15 days before a positive test (possible false negative), (ii) randomly sampled if they occurred within 15 days of each other (possibly during the same symptomatic illness), and (iii) randomly sampled if the individual contributed more than three negative tests during the study period. A stratum (defined by the regression equation as a unique combination of county and calendar week of testing) is considered analyzable if it includes at least one case and at least one control, because strata including only cases or only controls do not contribute to the estimation of the regression coefficients. For all cases and controls, all summarized characteristics correspond to only individuals who contributed at least one symptomatic test to an analyzable stratum.

**Characteristic**			**Stratified on Date of PCR Test**		**Stratified on Date of Vaccination**	
	**Underlying Population**<br><br>1+ dose of mRNA-1273 with no positive tests prior to first dose or in the first three days after first dose	**Eligible Population**<br><br>1+ symptomatic test at least four days after first dose	**Case Population**<br><br>1+ positive symptomatic test at least four days after first dose	**Control Population**<br><br>1+ negative symptomatic test at least four days after first dose, sub-sampled	**Case Population**<br><br>1+ positive symptomatic test at least four days after first dose	**Control Population**<br><br>1+ negative symptomatic test at least four days after first dose, sub-sampled
Number of individuals	143443	22864	2684	14885	2774	19485
Number of symptomatic tests						
Total		28916	2684	17691	2774	24864
Bucketed by time since full vaccination date						
- Days 4-10			155	309	205	686
- Days 11-13			33	148	41	315
- Days 14-20			54	348	54	706
- Days 21-27			32	363	27	659
- Days 28-34			24	338	25	606
- Days 35-41			3	243	4	489
- Days 42-71			23	804	22	1838
- Days 72-101			28	509	26	1619

- Days 102-131<br>- Days 132-161<br>- Days 162-191<br>- Days 192-221<br>- Days 222-251<br>- Days 252-281<br>- Days 282-311<br>- Days 312-341<br>- Days 342+			60<br>138<br>270<br>479<br>508<br>458<br>269<br>107<br>43	661<br>1488<br>2445<br>3451<br>3172<br>1986<br>900<br>402<br>124	57<br>141<br>274<br>484<br>519<br>466<br>275<br>110<br>44	1816<br>2305<br>2937<br>3749<br>3381<br>2160<br>1026<br>444<br>128
Age (years)						
- Mean (sd)	57.1 (18.3)	55.8 (18.5)	52.6 (17.0)	54.3 (18.5)	52.8 (17.1)	56.2 (18.5)
- 18-24	8,713 (6.1%)	1,391 (6.1%)	142 (5.3%)	1,004 (6.7%)	149 (5.4%)	1,150 (5.9%)
- 25-34	13,239 (9.2%)	2,494 (10.9%)	325 (12.1%)	1,816 (12.2%)	328 (11.8%)	2,100 (10.8%)
- 35-44	16,343 (11.4%)	3,096 (13.5%)	508 (18.9%)	2,180 (14.6%)	520 (18.7%)	2,584 (13.3%)
- 45-54	18,542 (12.9%)	3,143 (13.7%)	463 (17.3%)	2,153 (14.5%)	473 (17.1%)	2,629 (13.5%)
- 55-64	28,447 (19.8%)	4,394 (19.2%)	519 (19.3%)	2,831 (19.0%)	536 (19.3%)	3,713 (19.1%)
- 65-74	36,001 (25.1%)	5,125 (22.4%)	489 (18.2%)	3,097 (20.8%)	514 (18.5%)	4,474 (23.0%)
- 75-84	16,106 (11.2%)	2,149 (9.4%)	174 (6.5%)	1,180 (7.9%)	183 (6.6%)	1,877 (9.6%)
- 85+	5,631 (3.9%)	1,072 (4.7%)	64 (2.4%)	624 (4.2%)	71 (2.6%)	958 (4.9%)
State of Residence						
- Arizona	11,430 (8.0%)	961 (4.2%)	101 (3.8%)	591 (4.0%)	102 (3.7%)	830 (4.3%)
- Florida	27,764 (19.4%)	3,386 (14.8%)	330 (12.3%)	2,226 (15.0%)	377 (13.6%)	3,132 (16.1%)
- Iowa	3,799 (2.6%)	211 (0.9%)	13 (0.5%)	20 (0.1%)	21 (0.8%)	63 (0.3%)
- Minnesota	71,616 (49.9%)	12,786 (55.9%)	1,658 (61.8%)	8,756 (58.8%)	1,667 (60.1%)	10,959 (56.2%)
- Wisconsin	19,840 (13.8%)	5,423 (23.7%)	582 (21.7%)	3,292 (22.1%)	604 (21.8%)	4,498 (23.1%)
- Other	8,994 (6.3%)	97 (0.4%)	0 (0.0%)	0 (0.0%)	3 (0.1%)	3 (0.0%)
Sex						
- Female	76,932 (53.6%)	13,601 (59.5%)	1,579 (58.8%)	8,977 (60.3%)	1,631 (58.8%)	11,627 (59.7%)
- Male	66,498 (46.4%)	9,262 (40.5%)	1,104 (41.1%)	5,907 (39.7%)	1,142 (41.2%)	7,857 (40.3%)
- Unknown	13 (0.0%)	1 (0.0%)	1 (0.0%)	1 (0.0%)	1 (0.0%)	1 (0.0%)
Race						
- Asian	3,145 (2.2%)	488 (2.1%)	36 (1.3%)	352 (2.4%)	37 (1.3%)	453 (2.3%)
- Black	4,040 (2.8%)	473 (2.1%)	52 (1.9%)	314 (2.1%)	52 (1.9%)	399 (2.0%)
- Native American	604 (0.4%)	86 (0.4%)	8 (0.3%)	60 (0.4%)	8 (0.3%)	80 (0.4%)
- Hawaiian/Pacific	134 (0.1%)	17 (0.1%)	0 (0.0%)	10 (0.1%)	1 (0.0%)	16 (0.1%)
- White	128,384 (89.5%)	21,262 (93.0%)	2,504 (93.3%)	13,788 (92.6%)	2,591 (93.4%)	18,091 (92.8%)
- Other	2,686 (1.9%)	385 (1.7%)	56 (2.1%)	264 (1.8%)	56 (2.0%)	324 (1.7%)
- Unknown	4,450 (3.1%)	153 (0.7%)	28 (1.0%)	97 (0.7%)	29 (1.0%)	122 (0.6%)
Ethnicity: Hispanic/Latino						
- Yes	5,780 (4.0%)	863 (3.8%)	119 (4.4%)	583 (3.9%)	126 (4.5%)	724 (3.7%)
- No	131,020 (91.3%)	21,713 (95.0%)	2,527 (94.2%)	14,116 (94.8%)	2,609 (94.1%)	18,509 (95.0%)
- Unknown	6,643 (4.6%)	288 (1.3%)	38 (1.4%)	186 (1.2%)	39 (1.4%)	252 (1.3%)
Comorbidities						
- Cancer	7,423 (6.2%)	1,374 (6.3%)	108 (4.2%)	836 (5.9%)	112 (4.2%)	1,240 (6.6%)
- Cardiovascular	26,708 (22.4%)	6,053 (27.6%)	585 (22.8%)	3,767 (26.4%)	614 (23.1%)	5,318 (28.4%)
- Diabetes	8,326 (7.0%)	2,037 (9.3%)	201 (7.8%)	1,246 (8.7%)	217 (8.2%)	1,786 (9.5%)
- HIV/AIDS	71 (0.1%)	16 (0.1%)	1 (0.0%)	10 (0.1%)	2 (0.1%)	14 (0.1%)
- Kidney disease	7,055 (5.9%)	1,650 (7.5%)	146 (5.7%)	965 (6.8%)	156 (5.9%)	1,459 (7.8%)
- Liver disease	4,762 (4.0%)	1,068 (4.9%)	102 (4.0%)	699 (4.9%)	114 (4.3%)	943 (5.0%)
- Obesity	13,543 (11.4%)	3,545 (16.2%)	408 (15.9%)	2,221 (15.6%)	424 (15.9%)	3,067 (16.4%)
- Pulmonary	10,552 (8.9%)	2,809 (12.8%)	267 (10.4%)	1,817 (12.7%)	275 (10.3%)	2,493 (13.3%)
Dates of first vaccine dose						
- Earliest	12/2/20	12/20/20	12/21/20	12/20/20	12/21/20	12/20/20

- 25th %<br>- Median<br>- 75th %<br>- Latest	2/3/21<br>2/28/21<br>3/26/21<br>1/31/22	2/2/21<br>2/24/21<br>3/21/21<br>12/21/21	2/3/21<br>3/2/21<br>3/25/21<br>12/20/21	2/2/21<br>2/26/21<br>3/23/21<br>12/20/21	2/1/21<br>2/26/21<br>3/24/21<br>12/20/21	1/29/21<br>2/19/21<br>3/18/21<br>12/15/21
Days between first dose and test<br>- Minimum<br>- 25th %<br>- Median<br>- 75th %<br>- Maximum		 4<br>96<br>182<br>233<br>370	 4<br>177<br>224<br>264<br>368	 4<br>147<br>202<br>241<br>370	 4<br>174<br>223<br>264<br>368	 4<br>89<br>176<br>229<br>367

Table S5. Adjusted odds of symptomatic SARS-CoV-2 infection, non-COVID-19 hospitalization, and non-COVID-19 pneumonia for all non-strata covariates except time since vaccination in individuals who received at least one dose of mRNA-1273, derived from the CLR with stratification on the calendar date of testing. In total, there were 2684 positive symptomatic tests, 4025 non-COVID-19 hospitalizations, and 310 cases of non-COVID-19 pneumonia which contributed to analyzable strata. Adjusted odds were estimated with linear spline equations for age or by exponentiating the coefficients derived from conditional logistic regression models fit separately for each outcome.

		**Adjusted Odds Ratio (95% CI)**		
**Covariate**	**Level/ Category**	**Symptomatic Infection [N = 2684 events]**	**Non-COVID-19 Hospitalization [N = 4025 events]**	**Non-COVID-19 Pneumonia [N = 310 events]**
Age	18	1 (Reference)	1 (Reference)	1 (Reference)
	25	0.9 (0.59-1.37)	1.75 (1-3.06)	1.84 (0.18-18.79)
	35	1.45 (1.05-2.01)	1.4 (0.89-2.19)	1.07 (0.16-7.39)
	45	1.63 (1.16-2.29)	1.91 (1.2-3.04)	1.04 (0.14-7.69)
	55	1.29 (0.92-1.81)	3.01 (1.93-4.69)	5.07 (0.79-32.57)
	65	1.5 (1.07-2.1)	3.95 (2.55-6.12)	4.92 (0.77-31.33)
	75	1.19 (0.83-1.69)	7.27 (4.69-11.27)	6.47 (1.01-41.41)
	85	1.31 (0.87-1.97)	14.79 (9.48-23.06)	12.99 (2-84.47)
Comorbidities	Cardiovascular disease	0.841 (0.735, 0.962)	1.1 (0.995, 1.21)	0.737 (0.501, 1.09)
	Pulmonary disease	0.82 (0.708, 0.95)	1.2 (1.09, 1.32)	2.97 (2.08, 4.25)
	Diabetes	0.973 (0.814, 1.16)	1.3 (1.17, 1.44)	1.16 (0.77, 1.75)
	Kidney disease	1.13 (0.922, 1.38)	1.17 (1.05, 1.3)	1.12 (0.747, 1.68)
	Liver disease	0.928 (0.746, 1.15)	1.4 (1.24, 1.59)	1.78 (1.12, 2.84)
	HIV/AIDS	0.94 (0.121, 7.29)	0.681 (0.237, 1.96)	1.96e-07 (0, Inf)
	Cancer	0.89 (0.719, 1.1)	0.98 (0.875, 1.1)	0.68 (0.434, 1.07)

	Obesity	1.16 (1.01, 1.33)	1.02 (0.924, 1.13)	0.728 (0.497, 1.07)
Race	White/Caucasian	1 (Reference)	1 (Reference)	1 (Reference)
	Asian	0.617 (0.44, 0.867)	1.26 (0.975, 1.62)	0.261 (0.0355, 1.92)
	Black/African American	0.954 (0.716, 1.27)	1.31 (1.02, 1.68)	2.01 (0.837, 4.81)
	Native American	0.751 (0.369, 1.53)	0.949 (0.503, 1.79)	0.867 (0.114, 6.61)
	Native Hawaiian / Pacific Islander	1.56e-06 (0, Inf)	0.681 (0.0923, 5.02)	7.31e-07 (0, Inf)
	Other	1.11 (0.824, 1.51)	1.05 (0.757, 1.46)	1.33 (0.381, 4.62)
	Unknown	1.33 (0.859, 2.07)	0.931 (0.492, 1.76)	2.35 (0.246, 22.4)
Ethnicity	Not Hispanic or Latino	1 (Reference)	1 (Reference)	1 (Reference)
	Hispanic or Latino	1.09 (0.88, 1.35)	0.958 (0.768, 1.19)	0.383 (0.114, 1.29)
	Unknown	1.13 (0.775, 1.65)	0.79 (0.525, 1.19)	0.367 (0.0697, 1.93)
Sex	Female	1 (Reference)	1 (Reference)	1 (Reference)
	Male	1.13 (1.04, 1.22)	1.22 (1.14, 1.31)	1.86 (1.45, 2.39)
	Unknown	2.01 (0.198, 20.4)	NA	NA

Table S6. Adjusted odds of experiencing each outcome of interest at defined dates following the first vaccine dose among individuals at least 65 years old. The outcomes of interest are symptomatic SARS-CoV-2 infection and non-COVID-19 associated hospitalization (negative control). The table shows the adjusted odds of experiencing each outcome at relevant time points after the first vaccine dose; the expected date of second dose administration is 28 days after the first dose, and the expected date of full vaccination is 42 days after the first dose. On the left side, results are shown for the conditional logistic regression (CLR) model stratified on residential county and the calendar date of SARS-CoV-2 testing. On the right side, results are shown for the CLR model stratified on residential county, the date of full vaccination, and the 7-day trailing county-level COVID-19 incidence on the date of the SARS-CoV-2 PCR test.

	Stratified on Date of Testing		*Stratified on Date of Vaccination*	
	**Symptomatic Infection**	**Non-COVID-19 Hospitalization**	**Symptomatic Infection**	**Non-COVID-19 Hospitalization**
**Days Since First Dose**	N = 727	N = 2640	N = 768	N = 2838
4	1 (Reference)	1 (Reference)	1 (Reference)	1 (Reference)
10	0.41 (0.11-1.47)	1.86 (0.7-4.97)	0.32 (0.08-1.27)	1.53 (0.76-3.08)
14	0.43 (0.14-1.38)	1.32 (0.6-2.89)	0.53 (0.19-1.46)	1.04 (0.6-1.83)
28	0.15 (0.05-0.52)	1.45 (0.67-3.14)	0.11 (0.03-0.38)	1.37 (0.79-2.36)
42	0.04 (0.01-0.18)	1.38 (0.65-2.95)	0.06 (0.01-0.27)	1.45 (0.86-2.44)
92	0.2 (0.04-1.14)	1.41 (0.65-3.1)	0.04 (0.01-0.21)	1.78 (1.05-3.01)
142	0.38 (0.1-1.42)	1.41 (0.63-3.15)	0.38 (0.13-1.1)	1.49 (0.87-2.53)
192	0.21 (0.06-0.7)	0.88 (0.39-1.97)	0.63 (0.25-1.61)	0.99 (0.58-1.71)
242	0.27 (0.08-0.89)	0.81 (0.36-1.82)	0.84 (0.32-2.17)	0.98 (0.56-1.72)
292	0.37 (0.11-1.22)	0.92 (0.4-2.11)	1.76 (0.67-4.62)	1.23 (0.68-2.2)

Table S7. Adjusted odds of symptomatic SARS-CoV-2 infection, non-COVID-19 hospitalization, and non-COVID-19 pneumonia for all non-strata covariates except time since vaccination in individuals who received at least one dose of mRNA-1273, derived from the CLR with stratification on the calendar date of vaccination. In total, there were 2774 positive symptomatic tests, 4286 non-COVID-19 hospitalizations, and 329 cases of non-COVID-19 pneumonia which contributed to analyzable strata. Adjusted odds were estimated with linear spline equations for age or by exponentiating the coefficients derived from conditional logistic regression models fit separately for each outcome.

		**Adjusted Odds Ratio (95% CI)**		
**Covariate**	**Level/ Category**	**Symptomatic Infection**<br>**[N = 2774 events]**	**Non-COVID-19 Hospitalization**<br>**[N = 4286 events]**	**Non-COVID-19 Pneumonia**<br>**[N = 329 events]**
Age	18	1 (Reference)	1 (Reference)	1 (Reference)
	25	0.79 (0.51-1.22)	1.88 (1.06-3.34)	1.89 (0.19-18.48)
	35	1.36 (0.97-1.92)	1.42 (0.89-2.26)	0.82 (0.12-5.57)
	45	1.5 (1.05-2.14)	1.96 (1.21-3.17)	0.96 (0.13-6.87)
	55	1.18 (0.83-1.68)	3.12 (1.96-4.96)	4.25 (0.67-26.85)
	65	1.37 (0.96-1.96)	4.3 (2.72-6.81)	4.24 (0.69-26.15)
	75	1.11 (0.76-1.62)	8.57 (5.41-13.57)	6.48 (1.03-40.77)
	85	1.34 (0.87-2.05)	15.28 (9.61-24.3)	9.66 (1.53-60.87)
Comorbidities	Cardiovascular disease	0.823 (0.717, 0.946)	1.09 (0.988, 1.2)	0.749 (0.508, 1.1)
	Pulmonary disease	0.814 (0.7, 0.948)	1.12 (1.02, 1.23)	2.57 (1.81, 3.66)
	Diabetes	1.04 (0.867, 1.24)	1.25 (1.13, 1.39)	1.18 (0.792, 1.75)
	Kidney disease	1.11 (0.901, 1.36)	1.14 (1.03, 1.26)	1.16 (0.784, 1.71)
	Liver disease	0.958 (0.767, 1.2)	1.34 (1.18, 1.52)	1.57 (0.991, 2.47)
	HIV/AIDS	2.97 (0.721, 12.3)	0.974 (0.382, 2.48)	1.32e-06 (0, Inf)
	Cancer	0.91 (0.736, 1.12)	1.04 (0.937, 1.17)	0.855 (0.557, 1.31)

	Obesity	1.17 (1.01, 1.35)	1.08 (0.976, 1.18)	0.8 (0.548, 1.17)
Race	White/Caucasian	1 (Reference)	1 (Reference)	1 (Reference)
	Asian	0.588 (0.414, 0.837)	1.18 (0.913, 1.52)	0.245 (0.0334, 1.79)
	Black/African American	0.903 (0.664, 1.23)	1.3 (1.01, 1.67)	1.52 (0.576, 4.03)
	Native American	0.613 (0.293, 1.28)	1.06 (0.588, 1.9)	0.974 (0.126, 7.51)
	Native Hawaiian / Pacific Islander	0.365 (0.0434, 3.06)	0.749 (0.0988, 5.68)	3.74e-06 (0, Inf)
	Other	1.14 (0.836, 1.56)	1.03 (0.747, 1.42)	1.9 (0.532, 6.77)
	Unknown	1.43 (0.911, 2.25)	0.778 (0.401, 1.51)	1.25 (0.117, 13.3)
Ethnicity	Not Hispanic or Latino	1 (Reference	1 (Reference)	1 (Reference)
	Hispanic or Latino	1.04 (0.84, 1.29)	0.989 (0.793, 1.23)	0.351 (0.101, 1.22)
	Unknown	1.08 (0.732, 1.6)	0.826 (0.549, 1.24)	0.591 (0.111, 3.15)
Sex	Female	1 (Reference)	1 (Reference)	1 (Reference)
	Male	1.11 (1.02, 1.21)	1.24 (1.16, 1.32)	2.07 (1.61, 2.66)
	Unknown	0.878 (0.0765, 10.1)	NA	NA
Dominant variant	Alpha	1 (Reference)	1 (Reference)	1 (Reference)
	Delta	0.867 (0.605, 1.24)	0.912 (0.766, 1.09)	0.995 (0.514, 1.92)
	Omicron	1.41 (0.943, 2.1)	0.757 (0.504, 1.14)	0.21 (0.0396, 1.12)
	Neither	1.08 (0.749, 1.55)	0.968 (0.799, 1.17)	0.975 (0.453, 2.1)
	Unknown	1.17 (0.845, 1.61)	1.28 (1.1, 1.48)	0.952 (0.536, 1.69)

Table S8. Summary statistics for conditional logistic regression models. Each model is defined by a unique combination of Stratification Type (date of testing versus date of vaccination), reference timepoint (first dose versus full vaccination), and outcome (symptomatic infection versus non-COVID-19 hospitalization versus non-COVID-19 pneumonia. For each model, the Nagelkerke R-squared value (also known as a pseudo R-squared value) is shown along with the maximum R-squared value.

Stratification Type	Time Since First Dose or Full Vax	Outcome	Pseudo-R^2	Max R^2
Date of PCR Test	First Dose	Non-COVID-19 hospitalization	0.098	0.643
Date of PCR Test	First Dose	Non-COVID-19 pneumonia	0.032	0.203
Date of PCR Test	First Dose	Symptomatic SARS-CoV-2 infection	0.022	0.585
Date of PCR Test	Full Vax	Non-COVID-19 hospitalization	0.095	0.634
Date of PCR Test	Full Vax	Non-COVID-19 pneumonia	0.032	0.206
Date of PCR Test	Full Vax	Symptomatic SARS-CoV-2 infection	0.008	0.597
Date of Vaccination	First Dose	Non-COVID-19 hospitalization	0.081	0.659
Date of Vaccination	First Dose	Non-COVID-19 pneumonia	0.012	0.098
Date of Vaccination	First Dose	Symptomatic SARS-CoV-2 infection	0.026	0.458
Date of Vaccination	Full Vax	Non-COVID-19 hospitalization	0.078	0.647
Date of Vaccination	Full Vax	Non-COVID-19 pneumonia	0.013	0.102
Date of Vaccination	Full Vax	Symptomatic SARS-CoV-2 infection	0.022	0.466

Table S9. Variance inflation factors (VIFs) for conditional logistic regression covariates. VIFs are used to assess multicollinearity between independent variables in each regression model. A VIF greater than or equal to 5 was considered as evidence that the given covariate showed a concerning degree of multicollinearity with at least one other variable.

	Stratified on Date of PCR Test		Stratified on Date of Vaccination	
**Covariate**	**Time Since First Dose**	**Time Since Full Vax**	**Time Since First Dose**	**Time Since Full Vax**
Age	1.2	1.2	1.25	1.25
Comorbidity - Cancer	1.17	1.17	1.18	1.18
Comorbidity - Cardiovascular disease	2.01	2	2.08	2.09
Comorbidity - Diabetes	1.45	1.44	1.46	1.46
Comorbidity - HIV / AIDS	1	1	1	1
Comorbidity - Kidney disease	1.34	1.32	1.36	1.36
Comorbidity - Liver disease	1.16	1.16	1.16	1.16
Comorbidity - Obesity	1.54	1.54	1.56	1.56
Comorbidity - Pulmonary disease	1.32	1.32	1.32	1.33
County-level COVID-19 cases per 100K	NA	NA	1.49	1.61
Days since 1st vaccine dose	1	NA	2.72	NA
Days since 2nd vaccine dose	NA	1.01	NA	2.4
Ethnicity - Hispanic or Latino	1.2	1.19	1.2	1.18
Ethnicity - Unknown	1.26	1.25	1.26	1.23
Race - Asian	1.01	1.01	1.01	1.01
Race - Black / African American	1.01	1.01	1.01	1.01
Race - Native American	1	1	1	1
Race - Native Hawaiian / Pacific Islander	1	1.01	1	1
Race - Other	1.18	1.16	1.18	1.16
Race - Unknown	1.26	1.26	1.26	1.23
Sex - Male	1.02	1.03	1.02	1.02
Variant - Delta	NA	NA	4.11	3.93
Variant - Neither	NA	NA	1.29	1.14
Variant - Omicron	NA	NA	1.62	1.84
Variant - Uncertain	NA	NA	1.9	2.15
